# A systematic review and meta-analysis of the clinimetric properties of the core outcome measurement instruments for clinical effectiveness trials of nutritional and metabolic interventions in critical illness (CONCISE)

**DOI:** 10.1186/s13054-023-04729-7

**Published:** 2023-11-20

**Authors:** T. W. Davies, E. Kelly, R. J. J. van Gassel, M. C. G. van de Poll, J. Gunst, M. P. Casaer, K. B. Christopher, J. C. Preiser, A. Hill, K. Gundogan, A. Reintam-Blaser, A.-F. Rousseau, C. Hodgson, D. M. Needham, S. J. Schaller, T. McClelland, J. J. Pilkington, C. M. Sevin, P. E. Wischmeyer, Z. Y. Lee, D. Govil, L. Chapple, L. Denehy, J. C. Montejo-González, B. Taylor, D. E. Bear, R. M. Pearse, A. McNelly, J. Prowle, Z. A. Puthucheary

**Affiliations:** 1 Faculty of Medicine & Dentistry, Queen Mary University of London, London, EC1M 6BQ UK; 2 Critical Care and Perioperative Medicine Research Group, Adult Critical Care Unit, Royal London Hospital, London, E1 1BB UK; 3 Department of Intensive Care Medicine, School of Nutrition and Translational Research in Metabolism (NUTRIM), Maastricht University Medical Centre+, Maastricht, The Netherlands; 4 Department of Surgery, School of Nutrition and Translational Research in Metabolism (NUTRIM), Maastricht University Medical Centre+, Maastricht, The Netherlands; 5 Clinical Department and Laboratory of Intensive Care Medicine, Department of Cellular and Molecular Medicine, KU Leuven, Herestraat 49, 3000 Louvain, Belgium; 6 Division of Renal Medicine, Channing Division of Network Medicine, Brigham and Women’s Hospital, Boston, MA USA; 7 Medical Direction, Erasme University Hospital, Universite Libre de Bruxelles, Brussels, Belgium; 8 Department of Intensive Care Medicine, University Hospital RWTH, 52074 Aachen, Germany; 9 Department of Anesthesiology, University Hospital RWTH, 52074 Aachen, Germany; 10 Division of Intensive Care Medicine, Department of Internal Medicine, Erciyes University School of Medicine, Kayseri, Turkey; 11 Department of Anaesthesiology and Intensive Care, University of Tartu, Tartu, Estonia; 12 Department of Intensive Care Medicine, Lucerne Cantonal Hospital, Lucerne, Switzerland; 13 Department of Intensive Care, University Hospital of Liège, Liege, Belgium; 14 Australian and New Zealand Intensive Care Research Centre, School of Public Health and Preventive Medicine, Monash University, 3/553 St Kilda Rd, Melbourne, VIC 3004 Australia; 15 Department of Intensive Care and Hyperbaric Medicine, The Alfred, Melbourne, VIC Australia; 16 Outcomes After Critical Illness and Surgery (OACIS) Research Group, Johns Hopkins University, Baltimore, MD USA; 17 Pulmonary and Critical Care Medicine, Department of Medicine, and Department of Physical Medicine and Rehabilitation, Johns Hopkins University School of Medicine, Baltimore, MD USA; 18 Department of Anesthesiology and Intensive Care Medicine (CVK, CCM), Charité - Universitätsmedizin Berlin, Corporate Member of Freie Universität Berlin, Humboldt-Universität Zu Berlin, Berlin Institute of Health, Berlin, Germany; 19 Department of Anesthesiology and Intensive Care, School of Medicine, Klinikum Rechts Der Isar, Technical University of Munich, Munich, Germany; 20 Centre for Bioscience, Manchester Metropolitan University, John Dalton Building, Chester Street, Manchester, UK; 21 Department of Medicine, Division of Allergy, Pulmonary, and Critical Care Medicine, Vanderbilt University Medical Center, Nashville, TN USA; 22 Department of Anesthesiology, Duke University School of Medicine, DUMC, Box 3094 Mail # 41, 2301 Erwin Road, Durham, NC 5692 HAFS27710 USA; 23 Department of Anesthesiology, University of Malaya, Kuala Lumpur, Malaysia; 24 Department of Cardiac, Anesthesiology & Intensive Care Medicine, Charité, Berlin, Germany; 25 Institute of Critical Care and Anesthesia, Medanta: The Medicty, Gurugram, Haryana India; 26 Adelaide Medical School, Faculty of Health and Medical Sciences, The University of Adelaide, Adelaide, SA Australia; 27 School of Health Sciences, The University of Melbourne, Melbourne, Australia; 28 Department of Allied Health, Peter McCallum Cancer Centre, Melbourne, Australia; 29 Instituto de Investigación I+12, Hospital Universitario, 12 de Octubre, Madrid, Spain; 30 Department of Research for Patient Care Services, Barnes-Jewish Hospital, St. Louis, MO USA; 31 Department of Critical Care, Guy’s and St Thomas’ NHS Foundation Trust, London, UK; 32 Department of Nutrition and Dietetics, Guy’s and St Thomas’ NHS Foundation Trust, London, UK

**Keywords:** (3–5) Clinimetric, Core outcome set, Nutrition, Critical illness

## Abstract

**Background:**

CONCISE is an internationally agreed minimum set of outcomes for use in nutritional and metabolic clinical research in critically ill adults. Clinicians and researchers need to be aware of the clinimetric properties of these instruments and understand any limitations to ensure valid and reliable research. This systematic review and meta-analysis were undertaken to evaluate the clinimetric properties of the measurement instruments identified in CONCISE.

**Methods:**

Four electronic databases were searched from inception to December 2022 (MEDLINE via Ovid, EMBASE via Ovid, CINAHL via Healthcare Databases Advanced Search, CENTRAL via Cochrane). Studies were included if they examined at least one clinimetric property of a CONCISE measurement instrument or recognised variation in adults ≥ 18 years with critical illness or recovering from critical illness in any language. The COnsensus-based Standards for the selection of health Measurement INstruments (COSMIN) checklist for systematic reviews of Patient-Reported Outcome Measures was used. The Preferred Reporting Items for Systematic Reviews and Meta-Analyses were used in line with COSMIN guidance. The COSMIN checklist was used to evaluate the risk of bias and the quality of clinimetric properties. Overall certainty of the evidence was rated using a modified Grading of Recommendations, Assessment, Development and Evaluation approach. Narrative synthesis was performed and where possible, meta-analysis was conducted.

**Results:**

A total of 4316 studies were screened. Forty-seven were included in the review, reporting data for 12308 participants. The Short Form-36 Questionnaire (Physical Component Score and Physical Functioning), sit-to-stand test, 6-m walk test and Barthel Index had the strongest clinimetric properties and certainty of evidence. The Short Physical Performance Battery, Katz Index and handgrip strength had less favourable results. There was limited data for Lawson Instrumental Activities of Daily Living and the Global Leadership Initiative on Malnutrition criteria. The risk of bias ranged from inadequate to very good. The certainty of the evidence ranged from very low to high.

**Conclusions:**

Variable evidence exists to support the clinimetric properties of the CONCISE measurement instruments. We suggest using this review alongside CONCISE to guide outcome selection for future trials of nutrition and metabolic interventions in critical illness.

*Trial registration : *PROSPERO** (**CRD42023438187). Registered 21/06/2023.

**Supplementary Information:**

The online version contains supplementary material available at 10.1186/s13054-023-04729-7.

## Introduction

Functional decline and disability affect many survivors of critical illness and can be long-lasting [1]. Post-intensive care syndrome comprises physical, cognitive, and mental health impairments, which can result in adverse socioeconomic consequences and are recognised by patients, clinicians, and public sector organisations as a major public health issue [2, 3]. Muscle wasting occurs rapidly in critical illness and is the result of decreased protein synthesis, bioenergetic failure, and intramuscular inflammation [4–6]. Nutritional and metabolic interventions may be able to reverse these pathological changes, improving patient outcomes [7]. The variation in outcomes collected makes comparison between trials challenging, limiting future systematic reviews and meta-analyses [8, 9].

A methodological approach to address this issue is the creation of a Core Outcome Set (COS). This approach does not prevent researchers from evaluating additional outcomes, however, it provides the minimum standard ensuring that essential outcomes within a research area are consistently assessed using the same measurement instruments. Core outcome measures for clinical effectiveness of nutritional and metabolic interventions in critical illness (CONCISE) is an internationally agreed set of outcomes and measurement instruments for use at 30 and 90 days post enrolment, in nutritional and metabolic clinical research in critically ill adults [10]. The development of CONCISE involved a systematic review identifying outcome measures used in critical care nutrition trials and their clinimetric properties followed by a consensus process. The following measurement instruments were recommended: Short Form-36 Physical Component Score (SF-36 PCS) [11], 30 s sit-to-stand (30STS) [12], 6-min walk test (6MWT) [13], Short Physical Performance Battery (SPPB) [14], Barthel Index [15], Katz Index [16], Lawton Instrumental Activities of Daily Living (IADL) [17], Global Leadership Initiative on Malnutrition criteria (GLIM) [18] and handgrip strength (HGS) [19].

Clinicians and researchers using the measurement instruments recommended by CONCISE need to be aware of the clinimetric properties of these measurement instruments, to ensure valid and reliable research. Clinimetric or measurement properties refer to the quality of the measurement tool and the quality of its performance [20]. This systematic review and meta-analysis aimed to summarise and evaluate the clinimetric properties of the measurement instruments recommended in CONCISE.

## Methods

The review was registered on PROSPERO (CRD42023438187) on 21st June 2023. This study followed the COnsensus-based Standards for the selection of health Measurement INstruments (COSMIN) methodology for systematic reviews of Patient-Reported Outcome Measures (PROMs) [21]. This is reported in line with the Preferred Reporting Items for Systematic Reviews and Meta-Analyses (PRISMA) statement (Additional file 1: Table S1) [22], as recommended by the COSMIN guidelines as we await the combined PRISMA-COSMIN guideline [23].

### Search strategy and selection criteria

A search strategy was designed based on the search filter for finding studies on clinimetric properties, developed by Terwee et al. [24]. The search strategy is outlined in the Additional file 1*.* Four electronic databases (MEDLINE via Ovid, EMBASE via Ovid, CINAHL via Healthcare Databases Advanced Search, and CENTRAL via Cochrane) were searched. Databases were searched from inception to December 2022. Studies identified in the preliminary systematic review process for CONCISE were added [8, 10]. Reference lists were manually searched to screen for eligible studies and relevant review articles. No limits for language, date or geographical region were used. Citations were imported to the web-based collaboration software platform, Covidence [25].

### Inclusion and exclusion criteria

Inclusion and exclusion criteria were established prior to screening. Studies were included if they examined at least one clinimetric property of a CONCISE measurement instrument in adults ≥ 18 years with critical illness or recovering from critical illness in any language. To ensure completeness, we also included studies examining the clinimetric properties of variations or components of CONCISE measurement instruments, including the Short Form-36 Physical Functioning (SF-36 PF), five times STS (5xSTS) and SPPB 4 m gait speed. We included systematic reviews and pooled analyses where they provided new data. Unpublished studies, preprints, and conference abstracts without subsequent study publication were excluded.

Two authors (TD, EK) screened each title and abstract independently to determine eligibility for inclusion. Disagreements were resolved through discussion with a third reviewer (ZP). Full texts were assessed by both authors against the predetermined inclusion and exclusion criteria. Data extraction was completed by two authors (TD, EK) independently using standardised extraction forms. Data extraction included publication details (e.g., title, year, journal), patient characteristics (e.g. age, sex, severity and duration of illness), details of measurement setting (e.g., type of intensive care unit (ICU), timeframe) and the predetermined clinimetric properties of the measurement instrument. Authors were contacted for missing demographic data. Clinimetric properties extracted were based on the COSMIN guidelines and are described in Table 1. Data included structural validity (factor analysis results on dimensionality), internal consistency (Cronbach’s alpha), reliability (intraclass correlations), measurement error (standard error of measurement (SEM), smallest detectable change (SDC) and minimal important change (MIC)), construct validity (convergent validity—correlation of CONCISE instruments with comparator measures (Additional file 1: Table S2), divergent validity—correlation of CONCISE instruments with dissimilar measures (Additional file 1: Table S2); and known-groups validity—comparison of CONCISE instrument scores between two subgroups using relative effect sizes or area under the curve (AUC)), responsiveness to change (mean differences, median differences, AUC or relative effect sizes), predictive validity (correlation, odds ratio, AUC or regression coefficient) and interpretability (floor and ceiling effects). Content validity (as per step 5 of COSMIN guidelines) [26] was not evaluated as the aim of this review was to present and evaluate the clinimetric properties of the measurement instruments which had reached consensus through rigorous methodology in CONCISE, and not to formulate additional recommendations about the use of specific outcome measurement instruments.

**Table 1:** COSMIN clinimetric properties and updated criteria for good measurement properties

Measurement property	Rating	Criteria
Structural validity *The degree to which the scores of an instrument are an adequate reflection of the dimensionality of the construct to be measure*	+	**CTT**: CFA: CFI or TLI or comparable measure > 0.95 OR RMSEA < 0.06 OR SRMR < 0.08^a^ EFA/PCA: Rotation method specified (e.g. varimax, promax, oblimin, etc.)^b^ *AND* Variance explained (total and/or per factor/component) reported^b^ IRT/Rasch: No violation of unidimensionality: CFI or TLI or comparable measure > 0.95 OR RMSEA < 0.06 OR SRMR < 0.08 *AND* no violation of local independence: residual correlations among the items after controlling for the dominant factor < 0.20 OR Q3's < 0.37 *AND* no violation of monotonicity: adequate looking graphs OR item scalability > 0.30 *AND* adequate model fit: **IRT**: χ2 > 0.01 **Rasch**: infit and outfit mean squares ≥ 0.5 and ≤ 1.5 OR Z-standardized values > ‐2 and < 2
	?	**CTT**: Not all information for ‘ + ’ reported **IRT/Rasch**: Model fit not reported
	–	Criteria for ‘ + ’ not met
Internal consistency *The degree of interrelatedness among items*	+	At least low evidence for sufficient structural validity AND Cronbach's alpha(s) ≥ 0.70 for each unidimensional scale or subscale
	?	Criteria for “At least low evidence for sufficient structural validity” not met
	–	At least low evidence for sufficient structural validity AND Cronbach’s alpha(s) < 0.70 for each unidimensional scale or subscale
Reliability *The extent to which scores for patients who have not changed are the same for repeated measurement under the following conditions: over time (test–retest); by different persons on the same occasion (inter-rater)*	+	ICC or weighted Kappa ≥ 0.70
	?	ICC or weighted Kappa not reported
	–	ICC or weighted Kappa < 0.70
Measurement error *The systematic and random error of a patient’s score that is not attributed to true changes in the construct to be measured*	+	SDC or LoA < MIC
	?	MIC not defined
	–	SDC or LoA > MIC
Hypothesis testing for construct validity *The degree to which the scores of an instrument are consistent with hypotheses, based on the assumption that the instrument validly measures the construct to be measured*	+	The result is in accordance with the hypothesis
	?	No hypothesis defined (by the review team)
	–	The result is not in accordance with the hypothesis
Responsiveness *The ability of an instrument to detect change over time in the construct to be measured*	+	The result is in accordance with the hypothesis OR AUC ≥ 0.70
	?	No hypothesis defined (by the review team)
	–	The result is not in accordance with the hypothesis OR AUC < 0.70
Interpretability *The degree to which one can assign qualitative meaning to an instrument’s quantitative scores or changes in scores*		Not applicable

χ^2^ chi-squared, *AUC* area under the curve, *CFA* confirmatory factor analysis, *CFI* comparative fit index, *CTT* classical test theory, *EFA* exploratory factor analysis, *ICC* intraclass correlation, *IRT* item response theory, *LoA* limits of agreement, *MIC* minimal important change, *PCA* principal component analysis, *RMSEA* root mean square error of approximation, *SDC* smallest detectable change, *SRMR* standardised root mean residual, *TLI* tucker-lewis index

^a^Hu and Bentler [84]

^b^Floyd and Widaman [85]

### Assessment of risk of bias and certainty of the evidence

Two independent reviewers (TD, EK) used the COSMIN checklist to evaluate the risk of bias of clinimetric properties, blinded to each other's ratings [21]. Disagreements were resolved by discussion with a third reviewer (ZP). Based on the risk of bias assessment, studies were rated as either very good, adequate, doubtful, or inadequate. Following this, each clinimetric property result was rated against the criteria for good measurement (clinimetric) properties (Table 1). Each result was rated as sufficient (+), insufficient (−) or indeterminate (?). Predictive validity was not rated as this is not included in the COSMIN checklist. Specific hypotheses were developed for construct validity and responsiveness (Additional file 1: Table S3). Construct validity and responsiveness were considered sufficient (+) if ≥ 75% of the hypotheses were met, or insufficient (−) if ≥ 75% of the hypotheses were not met, otherwise they were considered inconsistent (±) [21]. All results for each clinimetric property were qualitatively summarised and where appropriate, quantitatively pooled and this summarised result was evaluated against the criteria for good measurement (clinimetric) properties to get an overall rating. Finally, the evidence was graded using the modified Grading of Recommendations, Assessment, Development and Evaluation system approach (GRADE) approach [21]. GRADE was adopted and modified as per COSMIN guidelines to rate four of the five GRADE factors (risk of bias, inconsistency, imprecision, and indirectness). Disagreements were resolved by discussion with a third reviewer (ZP).

### Data synthesis

For reliability, where there were three or more studies, we calculated pooled intraclass correlation coefficients (ICCs) and 95% confidence intervals using a standard generic inverse variance random effects model. ICC values were combined based on estimates derived from a Fisher transformation, z = 0.5 × ln((1 + ICC)/(1 − ICC)), which has an approximate variance, (Var(z) = 1/(N-3)), where N is the sample size [27]. Between-study heterogeneity was evaluated using the I^2^ test. Where meta-analysis was not appropriate, we calculated weighted means (number of participants included per study) and weighted standard deviation. Where it was not possible to pool results statistically, results were descriptively summarised. Meta-analysis of data was performed using the statistical software package Review Manager 5.4 (RevMan 5.4.1). Where effect sizes were missing and studies provided sufficient data, Cohen's d was computed as the effect size to assess responsiveness. In cases where the data did not allow for Cohen's d calculation, standardised response mean (SRM) was used as an alternative effect size measure.

## Results

### Study selection

**Fig. 1:**
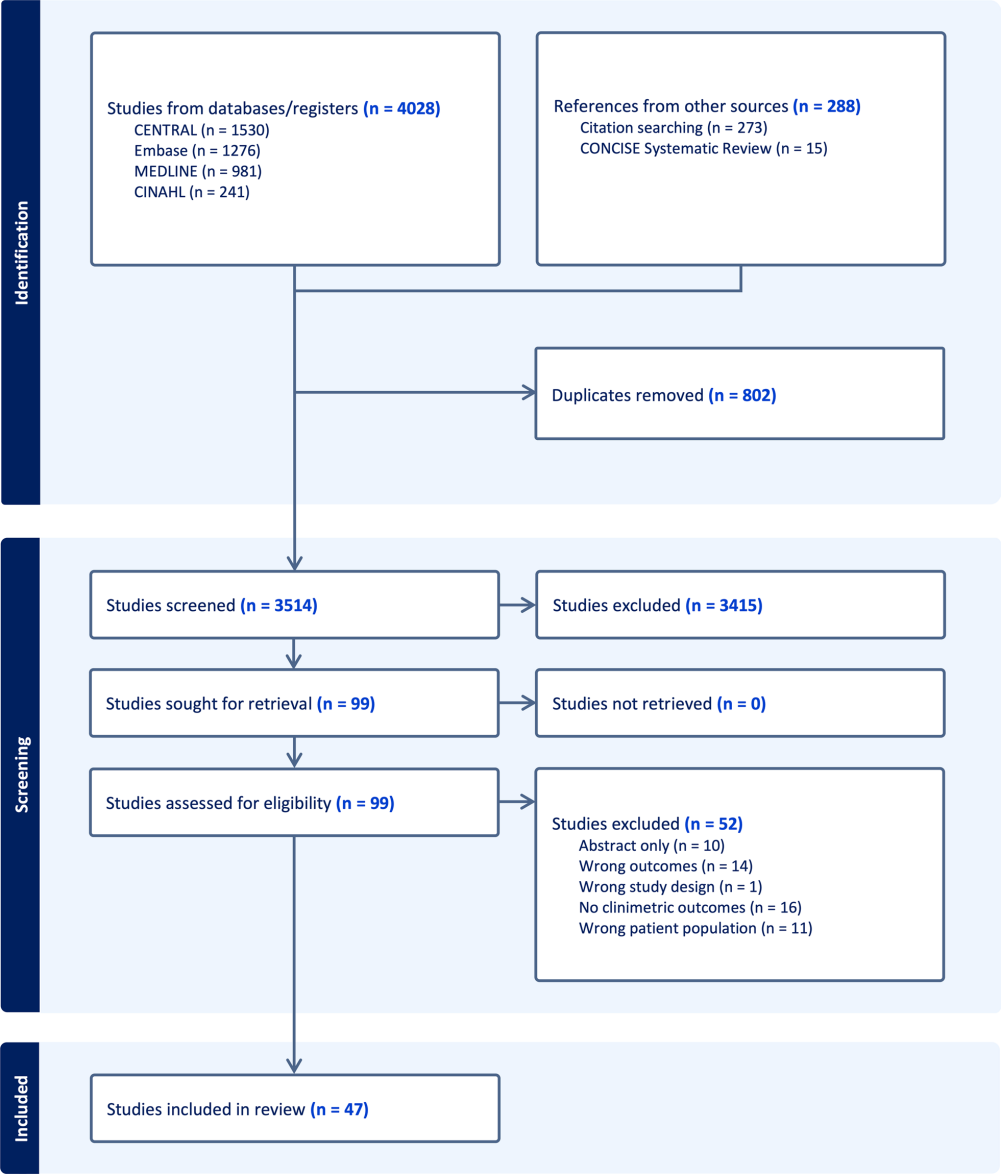
PRISMA diagram

The search identified 4316 studies. Forty-seven were included in the review, reporting data for 12,308 participants. PRISMA flow diagram is outlined in Fig. 1. All included articles were in English. Table 2 outlines the characteristics of the included studies.

**Table 2:** Key characteristics of included studies

Author, Year, Location	*n*	Gender M/F	Age mean ± SD or median [IQR]	Outcome Measure	Setting	Severity of illness APACHE ± SD or median [IQR]	ICU LOS (days) mean ± SD or median [IQR]	Timepoints of assessment for outcome of interest
Abd-El-Gawad et al., 2013, Egypt [55]	65	42/23	70 ± 11	KI	General ICU	NR	11 ± 8	1 month prior to ICU admission
Ali et al., 2008, USA [64]	136	65/71	58 ± 16	HGS	General ICU	66 ± 27	ICUAW 21 ± 19, non-ICUAW 12 ± 10	Ventilator day 5
Alison et al., 2012, Australia [28]	173	104/69	57 ± 16	SF-36 PF, 6MWT	Post ICU clinic	19 ± 10	9 ± 8	Post hospital discharge (weeks 1, 8, 26)
Bakhru et., 2018, USA [29]	36	19/17	65 [IQR 28]	SPPB, SF-36 PF, SF-36 PCS, HGS	General ICU, Post ICU clinic	29 [IQR 8.0]	NR	Post hospital discharge [30 days]
Baldwin et al., 2013, Australia [65]	17	10/7	78 [46–82]	HGS	General ICU	20 ± 5	18 [12–21]	ICU admission (day 13)
Bo et al., 2003, Italy [56]	659	352/307	77 ± 8	KI	General ICU	13 ± 5	7 ± 6	2 weeks prior to ICU admission
Broslawski et al.,1995, USA[63]	45	24/21	77 ± 8	KI	General ICU	19 ± 8	7 ± 8	6 months after ICU discharge
Bruno et al., 2022, Global [62]	2359	1670/689	Non-frailty/disability 75 ± 4, Frailty or disability 77 ± 6, Frailty & Disability 78 ± 5	KI	General ICU (COVID)	NR SOFA: Non-frailty/disability 5, Frailty or disability 6, Frailty & disability 8	NR	Prior to ICU admission
Chiang et al., 2006, Taiwan [51]	32	24/8	Control 79 [73–82, 84], Treatment 75 [63–80]	BI	Post ICU clinic	NR	NR	NR
Chan et al., 2015, Global [30]	651	368/226	Between 48–59	SF-36 PF, 6MWT	General ICU	Between 19–84	Between 9–19	NR
Chan et al., 2016, Global [50]	306	157/149	ATLOS = 48 ± 15, ICAP = 46 ± 13	SPPB – 4 m gait speed	General ICU	ATLOS = 25 ± 8, ICAP = 24 ± 8	ATLOS = 14 ± 11, ICAP = 18 ± 18	36-, 48-, and 60- month ICU follow up
Chan et al., 2017, Global [40]	233	123/110	ATLOS = 48 ± 14, ICAP = 49 ± 15	SF-36 PCS, 6MWT	General ICU	ATLOS = 25 ± 8 ICAP = 24 ± 8	ATLOS = 15 ± 12, ICAP = 18 ± 17	6, 12 months post ICU follow up
Chan et al., 2018, Global [31]	120	57/63	50 ± 15	SF-36 PF, 6MWT	General ICU	86 ± 28	15 ± 11	6, 12 months post onset ARDS
Chrispin et al., 1997, UK [32]	166	113/53	62 [59–64]	SF-36 PF	General ICU	10 [8–11]	1	ICU discharge
Clini et al., 2011, Italy [57]	77	46/31	75 ± 7	KI	Ventilator weaning unit	12 ± 4	51 [12–115]	Prior to admission to weaning unit [24 days]
Costigan et al., 2019, Canada [44]	40	19/21	62 ± 17	STS	Mixed ICU	20 [14–28]	7 [4–12]	ICU discharge
Cottereau et al., 2015, France [66]	84	45/39	66 [53–79]	HGS	General ICU	NR	13 [7–24]	At the time of SBT
Daubin et al., 2011, France [58]	100	65/35	79 ± 3	KI	General ICU	24 [18–30]	NR	ICU admission
de Azevedo et al., 2021, Brazil [41]	181	99/82	66 ± 19	SF-36 PCS	Mixed ICU	NR. SAPS 53 [44–68]	32	3, 6 months post randomisation
Denehy et al., 2014, Australia/USA [39]	177	113/64	60 [49–72]	STS, SF-36 PCS, 6MWT	General ICU	19 [16–23]	8 [6–14]	Post ICU discharge [3 months]
dos Reis et al., 2022, Brazil [52]	122	62/60	56 [47–66]	BI	General ICU	21 ± 8	7 [5–11]	ICU discharge (± 24 h)
Fan et al., 2014, USA [67]	222	123/99	49 [40–58]	HGS	Home/post ICU clinic	23 [19–28]	14 [10–23]	Post hospital discharge (3, 6, 12 & 24 months)
Hermans 2012, Belgium [68]	75	38/32	59 [52–71]	HGS	Mixed ICU	NR	22 [15–30]	Not specified
Heyland et al., 2000, Canada [33]	30	16/14	62 ± 14	SF-36 PF, SF-36 PCS	General ICU	22 ± 6	12 ± 9	Post ICU discharge [1 year]
Kaarola et al., 2004, Finland [34]	1099	659/440	54 [41–65]	SF-36 PF	General ICU	14 [9–18]	2 [1–6]	1–6 years post discharge from ICU
Kawakami et al., 2021, Japan [42]	192	125/67	74 [64–81]	SF-36 PCS	Mixed ICU	23 [18–28]	7 [5–14]	Baseline, 6 months post ICU
Khoudri et al., 2007, Morocco [35]	145	79/66	38 ± 17	SF-36 PF	General ICU	14 ± 6	9 ± 7	3 months post ICU discharge
Lee et al., 2012, USA [69]	107	59/48	61 ± 18	HGS	Mixed ICU	15 ± 9	5 [3–10]	48 h after admission (or when able, median time 3 days)
Melo et al., 2019, Brazil [46]	96	48/48	62 ± 1	STS	General ICU	SAPS 32 ± 9	5 ± 2	Discharge from ICU to ward [3 times on the same day]
Melo et al., 2022, Brazil [47]	142	75/67	51 [43–64]	STS	General ICU	SAPS 40 [36–57]	3 [2–5]	ICU Discharge
Mohamed-Hussein et al., 2018, Egypt [70]	34	18/16	61 ± 12	HGS	Respiratory ICU	NR	10 ± 9	First 24 h after recovery from sedation, repeated every 24 h for 5 days
Needham et al., 2014, USA [36]	203	100/103	48 ± 15	SF-36 PF, 6MWT	Post ICU clinic	85 ± 25	14 ± 11	12 months after ALI onset
O'Grady et al., 2022, Global [45]	451	278/173	Between 60–66	STS	General ICU	Between 19–24	9 [7–11]	ICU discharge, hospital discharge
Parry et al., 2015, Australia [49]	66	40/26	58 ± 17	6MWT	General ICU	21 ± 7	8 [5–15]	Awakening, Discharge
Parry, Berney et al., 2015, Australia [71]	60	35/25	69 [49–77]	HGS	General ICU	22 [18–29]	12 [8–20]	Awakening
Parry et al., 2021, Australia [13]	60	35/25	69 [49–77]	6MWT, HGS	General ICU	22	12 [8–10]	Day of awakening
Puthucheary et al., 2020, Germany [37]	159	107/49	61 [43–72]	SF-36 PF, SF-36 PCS	General ICU	NR	28 [10–48]	6, 12, 24 months
Rosa et al., 2020, Brazil [48]	32	14/18	59 [38–68]	6MWT	Post ICU clinic	NR	7 [4–11]	Post ICU discharge (4 months)
Sacanella et al., 2009, Spain [53]	230	140/90	75 ± 6	BI, IADL	General ICU	20 ± 6	12 ± 12	ICU admission
Shahbazi et al., 2021. Iran [73]	109	58/51	61 ± 14	GLIM	General ICU (COVID)	15 ± 4	NR	First 48 h following ICU admission
Theilla et al., 2021. Israel [74]	84	58/26	51 ± 21	GLIM	General ICU	21 ± 8	7 ± 10	First 24 h following ICU admission
Tripathy et al., 2014, India [59]	109	80/29	75 ± 8	KI	Post ICU clinic	19 ± 7	7 ± 3	Baseline, 28 days post-admission and 12 months post-hospital discharge
Van Der Schaaf et al., 2008, Netherlands [54]	69	43/26	60 [19–71]	BI, HGD	General ICU	16 [12–20]	7 [5–17]	Post ICU discharge (3–5 days)
Vest et al., 2011, USA [60]	309	145/164	75 ± 9	KI	General ICU	24 ± 6	5 ± 6	One month and one year after ICU discharge
Weinert et al., 1997, USA [43]	24	16/8	40 ± 12	SF-36 PCS	General ICU	NR Lung injury score 2 ± 1	NR	6 months post-acute lung injury
Wischmeyer, 2017, USA [38]	125	60/65	111 ± 18	SF-36 PF, SF-36 PCS, 6MWT	General ICU	21 ± 7	18 [8–18]	3–6 months post-randomisation
Wu et al., 1995, USA [61]	2313	1295/1018	62	KI	General ICU	NR	NR	2 months post ICU admission

*APACHE* acute physiology and chronic health evaluation; ARDS = Acute Respiratory Distress Syndrome; *BI* Barthel index, *GLIM* global leadership initiative on malnutrition, *HGS* hand grip strength, *IADL* instrumental activities of daily living, *ICU* intensive care unit, *ICUAW* ICU acquired weakness, *IQR* inter quartile range, *KI* katz index, *LOS* length of stay, *NR* Not reported, *SAPS* simplified acute physiology score, *SBT* spontaneous breathing trial, *SF-36 PCS* short form-36 physical component score, *SF-36 PF* short form-36 physical functioning, *SOFA* sequential organ failure assessment, *SPPB* short physical performance battery, *STS* Sit-to-stand, *6MWT* 6-min walk test

### Risk of bias

The COSMIN risk of bias rating varied from inadequate to very good. Ratings for individual studies are provided in Additional file 1: Table S4*.* Multiple studies tested more than one measurement property (n = 15)*.* The breakdown of studies reporting clinimetric properties was as follows: structural validity (n = 0), internal consistency (n = 4), reliability (n = 10), measurement error (n = 9), hypothesis testing for construct validity (n = 25) and responsiveness (n = 12). Certainty of evidence was rated using the GRADE approach [21]. Ratings ranged from very low to high. GRADE ratings are outlined in Additional file 1: Table S5.

### Measurement instruments

**Fig. 2:**
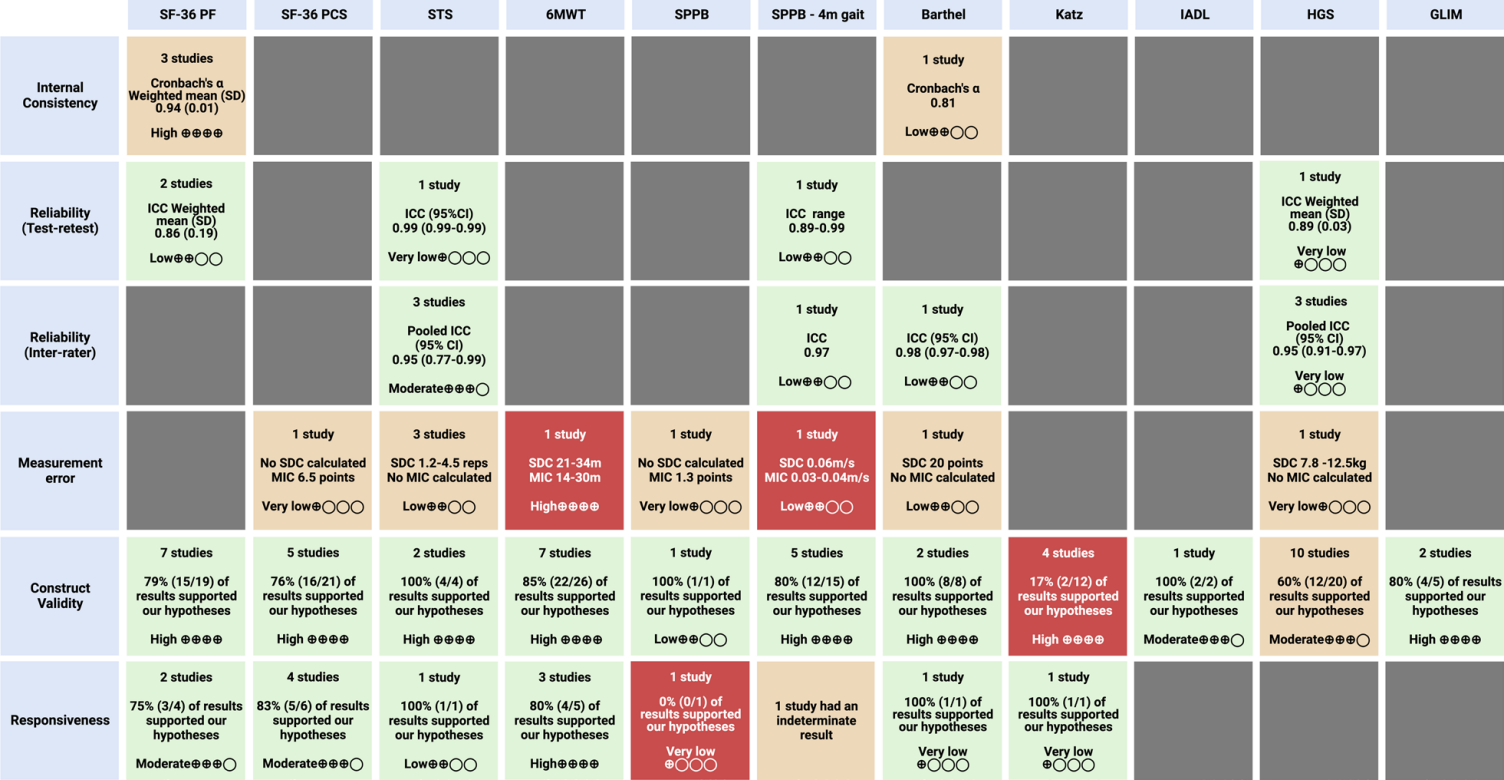
Results Overview. The colour of the box refers to the COSMIN criteria for good measurement (clinimetric) properties: Green = sufficient; orange = indeterminate or inconsistent; red = insufficient. The Grading of Recommendations, Assessment, Development and Evaluation System (GRADE) rating for the certainty of evidence is presented in each box. CI = confidence intervals; GLIM = global leadership initiative on malnutrition; HGS = handgrip strength; IADL = instrumental activities of daily living; ICC = intra class coefficient; MIC = minimal important change; SD = standard deviation; SDC = smallest detectable change; SF-36 PCS = short form-36 physical component score; SF-36 PF = short form-36 physical functioning; SPPB = short physical performance battery; STS = sit-to-stand; 6MWT = 6-min walk test

Full results are outlined in Additional file 1: Tables S6, S7, S8 and Fig. 2. No studies tested structural validity and it is therefore not included below.

### Physical function

#### Short Form-36 Physical Function (SF-36 PF)

**Fig. 3:**
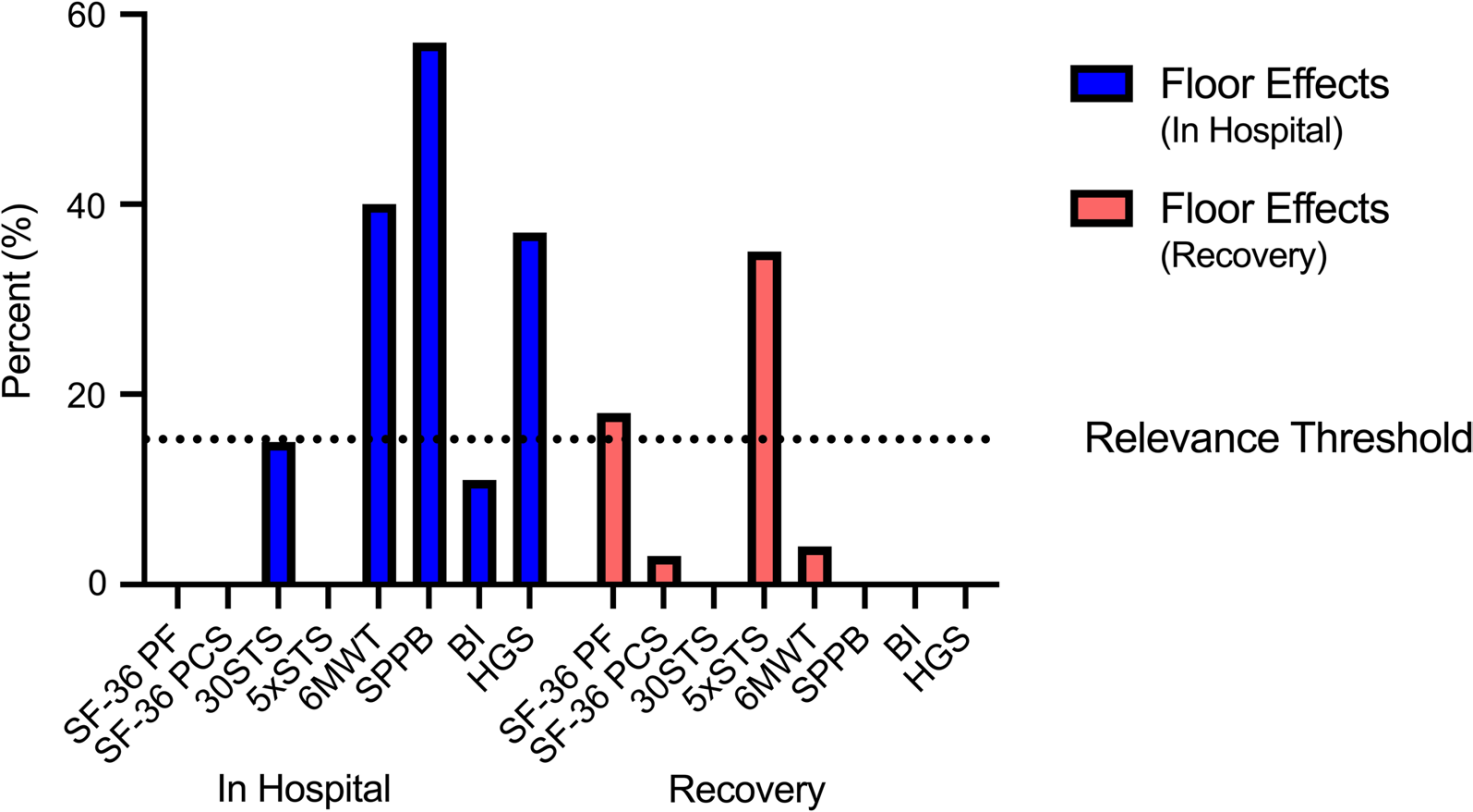
Floor effects in hospital and during recovery from critical illness. Floor effects for CONCISE measurement instruments in hospital and during recovery from critical illness. Where more than one study reported a result, the mean was calculated. Relevance threshold set at 15%. BI = barthel index; HGS = handgrip strength; SF-36 PCS = physical component score of the short form-36; SF-36 PF = physical functioning score of the short form-36; SPPB = short physical performance battery; 30STS = 30 s sit-to-stand; 5xSTS = five times sit-to-stand; 6MWT = 6-min walk test.

Eleven studies reported data for the SF-36 PF [28–38]. The SF-36 PF had excellent internal consistency (pooled Cronbach’s α 0.94) supported by a high certainty of evidence but was rated indeterminate due to no information on its structural validity. It had sufficient test–retest reliability (Pooled ICC 0.86) supported by a low certainty of evidence [32, 33, 35]. There was a moderate to high certainty of evidence supporting sufficient construct validity and responsiveness [29–31, 35–39]. No studies tested measurement error. Floor effects post ICU discharge ranged from 6 to 32% and ceiling effects post ICU discharge ranged from 9 to 38% (Additional file 1: Table S7 and Fig. 3) [34, 35, 37]. The SF-36 PF score at 1 month post ICU discharge was not predictive of 1 year mortality or 6 month readmissions [52]. There was no data on the association with length of stay.

#### Short Form-36 Physical Component Score (SF-36 PCS)

Nine studies reported data for the SF-36 PCS [29, 33, 37–43]. No studies tested internal consistency or reliability. There was a moderate to high certainty of evidence supporting sufficient construct validity and responsiveness [33, 37–43]. The MIC of the SF-36 PCS was 6.5 but measurement error was rated indeterminate due to no calculation of SDC [42]. A floor effect of 3% was seen at 6 months post ICU discharge (Additional file 1: Table S7 and Fig. 3) [42]. The SF-36 PCS score at 1 month post discharge was not predictive of 1 year mortality or 6 month readmissions [29]. There was no data on the association with length of stay.

#### Sit-to-stand (STS)

Two studies reported data for the 30STS [44, 45] and three studies for the 5xSTS [39, 46, 47]. When pooled together, there was a very low certainty of evidence supporting excellent test–retest reliability (ICC 0.99) and inter-rater reliability (Pooled ICC 0.95) [44, 46, 47]. Sufficient construct validity was supported by a high certainty of evidence [39, 47] and one study demonstrated sufficient responsiveness with a low certainty of evidence [39]. Measurement error was indeterminate due to no calculation of MIC but the SEM of the 30STS ranged from 0.51 to 1.51 repetitions and the SDC ranged from 1.19 to 4.45 repetitions [29, 35]. No floor or ceiling effects were seen at hospital discharge [35]. A floor effect of 15% was seen at ICU discharge when using the 30STS and 35% at 3 months post discharge when using the 5xSTS (Additional file 1: Table S7 and Fig. 3) [39, 45]. STS performance at ICU discharge was predictive of hospital length of stay [47]. There was no data on the association with mortality or hospital readmissions.

#### 6-min walk test (6MWT)

Nine studies reported data for the 6MWT [13, 28, 30, 31, 36, 38, 39, 48]. No studies in our review tested the reliability of the 6MWT. Sufficient construct validity and responsiveness were supported by a high certainty of evidence [13, 28, 30, 31, 39, 40]. Measurement error was rated as insufficient with a high certainty of evidence as the range for MIC was estimated to be 14-30 m by anchor-based methods which was lower than the SDC of 21–34 m [37]. A floor effect of 40% was seen at hospital discharge and 4% at 3 months post ICU discharge (Additional file 1: Table S7 and Fig. 3) [38, 39]. 6MWT performance at 3 and 6 months post ICU discharge can predict 1 year mortality, and hospital readmissions [6, 12] [30]. There was no data on the association with length of stay.

#### Short Physical Performance Battery (SPPB)

Two studies reported data for the SPPB [29, 49]. No studies in our review tested the reliability of the SPPB. Sufficient construct validity supported by a low certainty of evidence was demonstrated in one study [49]. Responsiveness to change was insufficient from awakening to ICU discharge (ES 0.33) with a very low certainty of evidence [49]. Measurement error was indeterminate due to no calculation of MIC. The reported range of SDC was 1.3–1.5 points [49]. The SPPB had a significant floor effect of 83% at awakening and 57% at ICU discharge (Additional file 1: Table S7 and Fig. 3) [49]. SPPB performance at 1 month post ICU discharge was not predictive of 1 year mortality or 6 month readmissions [29]. There was no data on the association with length of stay.

#### Short Physical Performance Battery (SPPB)—4 m gait speed

Five studies reported data on the SPPB 4 m gait speed [30, 31, 36, 40, 50]. Excellent test–retest reliability of the SPPB 4 m gait speed was supported by a low certainty of evidence (ICC range 0.89–0.99) [50]. Sufficient construct validity was supported by a high certainty of evidence and responsiveness was indeterminate [30, 31, 36, 40, 50]. Measurement error was rated insufficient with a high certainty of evidence as the range for MIC was estimated to be 0.13–0.14 m/s by anchor-based methods which was lower than the SDC of 0.06 m/s [50]. No studies tested interpretability. SPPB 4 m gait speed performed at 6 months was predictive of hospital readmissions between 6 to 12 months [40]. There was no data on the association with mortality or length of stay.

### Activities of daily living

#### Barthel Index

Four studies reported data for the Barthel Index [51–54]. It showed sufficient inter-rater reliability (ICC 0.98) and good internal consistency (Cronbach’s α 0.81) supported by a low certainty of evidence but was rated indeterminate for internal consistency due to no information on structural validity [52]. Sufficient construct validity was supported by a high certainty of evidence [52, 54]. Sufficient responsiveness was demonstrated in a single study with a very low certainty of evidence [51]. Measurement error was rated as indeterminate due to no calculation of MIC. A floor effect of 11% and a ceiling effect of 1% were seen at ICU discharge with an SEM of 7.2 points and an SDC of 20 points (Additional file 1: Table S7 and Fig. 3) [52]. There was no data on the association with mortality, hospital readmissions, or length of stay.

#### Katz Index

Eight studies reported data for the Katz Index [40, 55–61]. No studies in our review examined the Katz Index in terms of internal consistency, reliability, measurement error and interpretability. Construct validity was rated insufficient with a high certainty of evidence [40, 57, 60, 61]. Responsiveness was sufficient in a single study with a very low certainty of evidence [57]. The Katz index score on ICU admission was predictive of short term (in-hospital to 90 days) mortality but there was no data on the association with longer term mortality, hospital readmissions or length of stay [55, 56, 59, 62].

#### Instrumental Activities of Daily Living (Lawson IADL)

Four studies provided data on Lawson IADL [40, 53, 56, 63]. No studies in our review examined the IADL in terms of internal consistency, reliability, responsiveness, measurement error and interpretability. Sufficient construct validity was supported by a moderate certainty of evidence [40]. The IADL at ICU admission was predictive of long term mortality but there were conflicting results regarding shorter term mortality and it was not predictive of hospital length of stay [53, 56, 63]. When performed at 6 months, it was not predictive of hospital readmissions between 6 and 12 months [40].

### Muscle/nerve function

#### Handgrip strength (HGS)

Fifteen studies reported data on HGS [29, 36, 40, 47, 52, 54, 64–71]. There was excellent inter-rater reliability (Pooled ICC 0.95) and good test–retest reliability (Pooled ICC 0.89) supported by a very low to low certainty of evidence [65, 68]. Construct validity was inconsistent and no studies tested responsiveness [31, 36, 40, 47, 52, 54, 64, 69, 71, 72]. Measurement error was indeterminate due to no calculation of MIC. The SEM ranged between 2.8 to 4.5 kg and SDC 7.8 to 12.5 kg [65]. Significant floor effects were seen during ICU admission ranging from 26 to 55% (Additional file 1: Table S7 and Fig. 3) [64, 69, 71]. Handgrip strength performed well in the diagnosis of ICU-acquired weakness with high sensitivity and specificity [64]. Handgrip strength during ICU admission was not predictive of in-hospital mortality, hospital length of stay or ICU length stay [69–71]. When performed at 1 month and 6 months post ICU discharge, handgrip strength was not predictive of 1 year mortality or hospital readmissions [29, 40].

### Nutritional status

#### Global Leadership Initiative on Malnutrition Criteria (GLIM)

Two studies reported data for the GLIM [73, 74]. No studies in our review examined the GLIM in terms of reliability, responsiveness, measurement error and interpretability. There was a high certainty of evidence supporting sufficient construct validity. Two studies validated the GLIM against the Subjective Global Assessment (SGA) demonstrating a high level of precision (AUC 0.85–0.93) and agreement (Kappa 0.85) [48, 49]. The GLIM at ICU admission was predictive of ICU mortality and hospital length of stay [73]. There was no data on its association with longer term mortality and hospital readmissions.

## Discussion

This systematic review and meta-analysis evaluated the clinimetric properties of the measurement instruments recommended in CONCISE [10]. The SF-36 PCS, SF-36 PF, STS, 6MWT and Barthel Index had the strongest clinimetric properties and certainty of evidence. The SPPB, Katz Index and handgrip strength had less favourable results. There was limited available data for the IADL and GLIM.

### Measurement instruments

The CONCISE measurement instruments are established and considered feasible to use during critical illness and its recovery. Our review highlighted differences between the instruments in the strength of clinimetric properties and performance at different time points. The ability to stand from sitting unaided is increasingly recognised by patients as playing a fundamental role in activities of daily living [75–77], and our data shows the STS to be an attractive functional independence test with minimal floor effects at ICU and hospital discharge when the repetition based 30STS is used. Our data also support previous findings regarding the 6MWT being a well-defined test for use in critical care nutrition research, post ICU discharge [13, 30]. ICU survivors experience profound disability with previous work demonstrating that only 40% could ambulate at 7 days after ICU discharge [78]. As a result, more complex outcome measures including the 6MWT, SPPB and the Physical Function in ICU Test (PFIT-S) are plagued by floor effects at ICU or hospital discharge as demonstrated in our data [13, 38, 79]. The properties of the SPPB in critically ill patients are poorly defined with a significant floor effect at ICU discharge. Interestingly the 4 m gait speed test, a component of the SPPB, had robust clinimetric properties post hospital discharge suggesting its role may be best utilised later in the recovery period.

The SF-36 and its PCS are widely reported in critical care rehabilitation trials [80] with well-established clinimetric properties [37]. While our data supports excellent construct validity and responsiveness of the SF-36 PCS with no significant floor or ceiling effects, we found no data describing its internal consistency or reliability. The closely related SF-36 PF domain had excellent internal consistency and reliability but patients with good recovery trajectories have significant ceiling effects unlike those with persistent impairment where significant floor effects are seen [37].

Measurement of activities of daily living was deemed essential in the CONCISE Delphi process. Our data suggest the Barthel Index has the current best clinimetric properties with more limited evidence for the Katz Index and IADL. Handgrip strength had excellent inter-rater reliability but studies with a larger sample size are needed to improve the certainty of evidence to allow generalisability in trials of critical illness and there are significant floor effects when used during ICU admission.

The GLIM criteria are a diagnostic tool for malnutrition rather than a patient-reported or performance-based measurement instrument. Reliability, responsiveness, and measurement error testing, as described elsewhere in this review are therefore less relevant for the GLIM criteria and have not been studied. It was seen to be highly accurate in diagnosing malnutrition in critical illness and showed excellent construct validity when compared to the SGA supporting its use in the ICU setting.

### Implications for outcome selection and future research

The paucity of relevant research and the difficulty of face-to-face assessments during recovery from critical illness make mandating measurement instruments challenging. The use of patient-reported questionnaires, such as the SF-36, or objective performance-based measurement instruments that can be feasibly administered at home via telemedicine, such as the STS [81, 82], may improve loss to follow-up and enable adequate analysis of interventions over recovery from critical illness.

It has previously been suggested that a single measurement instrument to evaluate functional outcomes cannot be used due to the presence of floor and ceiling effects at different time points, which we highlight above [49]. This means identifying change over time or change in response to an intervention is challenging. The repetition based 30STS has robust clinimetric properties and no floor and ceiling effects at hospital discharge making it an attractive measure of physical function for longitudinal nutrition studies in critical illness.

The strong interest in activities of daily living suggests the Katz Index and IADL require further evaluation in the critically ill population. It has previously been suggested that the Barthel Index is more suitable than the Katz Index for assessing patients after an ICU stay [84] and our analysis supports this recommendation. Additional clinimetric research is required for a more complete evaluation of IADL, handgrip strength and GLIM. Without further research, these instruments may be less attractive for future clinical trials involving patient care. Defining measurement error and responsiveness in more detail for all CONCISE measurement instruments will aid future trial design and sample size calculation.

### Strengths and limitations

This review followed the COSMIN methodology and a rigorous approach was taken to the evaluation of the quality and certainty of evidence using the COSMIN risk of bias checklist, COSMIN’s criteria for good measurement properties and the modified GRADE approach [21]. The most important limitations are the low number of high-quality studies and the possibility that relevant studies with clinimetric data were missed in our searches hence results should be interpreted with this in mind. This is especially true for responsiveness where studies used a CONCISE measurement instrument but failed to comment specifically on responsiveness and therefore did not appear in our search. To minimise this, we included all randomised controlled trials of nutrition in critical illness since 2000 from the preliminary CONCISE systematic review [8, 10] but studies with non-nutritional interventions using CONCISE measurement instruments may have been missed. Due to the small number of studies, we included all studies in this review regardless of the risk of bias and subgroup analysis was not performed. We also had to adapt the COSMIN methodology for PROMs to use for the CONCISE performance-based and diagnostic measurement instruments. The studies examined were heterogeneous with variable time points of measurement which were often different to the 30 day or 90 day fixed time points we recommend in CONCISE. Finally, there were no studies evaluating structural validity and the risk of bias was doubtful in many of the studies due to the small sample size or other important methodological flaws such as an inappropriate time interval between assessments when examining reliability. This reinforces the need for large high-quality clinimetric studies in critical illness.

## Conclusion

The CONCISE measurement instruments are established and feasible to administer during critical illness and its recovery. The SF-36 PF, SF-36 PCS, STS 6MWT, and Barthel Index had the strongest clinimetric properties and certainty of evidence. Further clinimetric research into all the CONCISE measurement instruments will improve outcome selection for future trials of nutrition and metabolic interventions in critical illness and enable greater generalisability of findings between studies. We suggest using this review alongside CONCISE to guide outcome selection for future trials of nutrition and metabolic interventions in critical illness.

### Supplementary Information

**Additional file 1.** Supplementary Information.

## Data Availability

The datasets used and/or analysed during the current study are available from the corresponding author upon reasonable request.

## Abbreviations

ADL: Activities of daily living
AUC: Area under the curve
COS: Core outcome set
COSMIN: COnsensus-based Standards for the selection of health Measurement Instruments
GLIM: Global Leadership on Malnutrition
GRADE: Grading of Recommendations, Assessment, Development and Evaluation
IADL: Instrumental activities of daily living
ICC: Intraclass correlation coefficient
ICU: Intensive care unit
MIC: Minimal important change
MRC: Medical Research Council
PFIT-s: The Physical Function in ICU Test
PRISMA: Preferred Reporting Items for Systematic Reviews and Meta-analyses
ROB: Risk of bias
SF-36: Short Form-36 Questionnaire
SF-36 PCS: Short Form-36 Questionnaire Physical Component Score
SF-36 PF: Short Form-36 Questionnaire Physical Functioning
SDC: Smallest detectable change
SEM: Standard error of measurement
SGA: Subjective Global Assessment
SPPB: Short Physical Performance Battery
SRM: Standardised response mean
STS: Sit-to-stand
30STS: 30 second sit-to-stand test
5xSTS: Five times sit-to-stand
6MWT: 6-minute walk test
