# Supplementary material for: A systematic review and meta-analysis of the clinimetric properties of the core outcome measurement instruments for clinical effectiveness trials of nutritional and metabolic interventions in critical illness (CONCISE)
Source: Crit Care. 2023 Nov 20;27:450. doi: 10.1186/s13054-023-04729-7 (PMC10662687; doi:10.1186/s13054-023-04729-7)
Supplement: Supplementary file 1 — Additional file 1. Supplementary Information. [file 13054_2023_4729_MOESM1_ESM.docx]

**Title:**

A systematic review and meta-analysis of the measurement properties of the core outcome measurement instruments for clinical effectiveness trials of nutritional and metabolic interventions in critical illness (CONCISE).

**Table of Contents**

| **Supplementary material** | **Page Number** |
| --- | --- |
| Supplementary material. Search Strategy | 1 |
| Supplementary material Table S1: PRISMA checklist | 2 |
| Supplementary material Table S2. List of Comparator Instruments used in Hypothesis Testing for Construct Validity | 5 |
| Supplementary material Table S3. Hypotheses for Construct Validity and Responsiveness | 7 |
| Supplementary material Table S4. COSMIN Risk of Bias Ratings | 10 |
| Supplementary material Table S5. Grading of Recommendations, Assessment, Development and Evaluation system approach (GRADE) ratings | 12 |
| Supplementary material Table S6. Results of Individual Studies | 15 |
| Supplementary material Table S7*.* Floor and Ceiling Effects | 47 |
| Supplementary material Table S8*.* Summary of Clinimetric Properties and Quality of Evidence | 49 |
| Supplementary material References | 55 |

Supplementary Material

*Search Strategy*

(instrumentation[sh] OR methods[sh] OR Validation Studies[pt] OR Comparative Study[pt] OR "psychometrics"[MeSH] OR psychometr*[tiab] OR clinimetr*[tw] OR clinometr*[tw] OR "outcome assessment (health care)"[MeSH] OR outcome assessment[tiab] OR outcome measure*[tw] OR "observer variation"[MeSH] OR observer variation[tiab] OR "Health Status Indicators"[Mesh] OR "reproducibility of results"[MeSH] OR reproducib*[tiab] OR "discriminant analysis"[MeSH] OR reliab*[tiab] OR unreliab*[tiab] OR valid*[tiab] OR coefficient[tiab] OR homogeneity[tiab] OR homogeneous[tiab] OR "internal consistency"[tiab] OR (cronbach*[tiab] AND (alpha[tiab] OR alphas[tiab])) OR (item[tiab] AND (correlation*[tiab] OR selection*[tiab] OR reduction*[tiab])) OR agreement[tiab] OR precision[tiab] OR imprecision[tiab] OR "precise values"[tiab] OR test–retest[tiab] OR (test[tiab] AND retest[tiab]) OR (reliab*[tiab] AND (test[tiab] OR retest[tiab])) OR stability[tiab] OR interrater[tiab] OR inter-rater[tiab] OR intrarater[tiab] OR intra-rater[tiab] OR intertester[tiab] OR inter-tester[tiab] OR intratester[tiab] OR intra-tester[tiab] OR interobserver[tiab] OR inter-observer[tiab] OR intraobserver[tiab] OR intra-observer[tiab] OR intertechnician[tiab] OR inter-technician[tiab] OR intratechnician[tiab] OR intra-technician[tiab] OR interexaminer[tiab] OR inter-examiner[tiab] OR intraexaminer[tiab] OR intra-examiner[tiab] OR interassay[tiab] OR inter-assay[tiab] OR intraassay[tiab] OR intra-assay[tiab] OR interindividual[tiab] OR inter-individual[tiab] OR intraindividual[tiab] OR intra-individual[tiab] OR interparticipant[tiab] OR inter-participant[tiab] OR intraparticipant[tiab] OR intra-participant[tiab] OR kappa[tiab] OR kappa’s[tiab] OR kappas[tiab] OR repeatab*[tiab] OR ((replicab*[tiab] OR repeated[tiab]) AND (measure[tiab] OR measures[tiab] OR findings[tiab] OR result[tiab] OR results[tiab] OR test[tiab] OR tests[tiab])) OR generaliza*[tiab] OR generalisa*[tiab] OR concordance[tiab] OR (intraclass[tiab] AND correlation*[tiab]) OR discriminative[tiab] OR "known group"[tiab] OR factor analysis[tiab] OR factor analyses[tiab] OR dimension*[tiab] OR subscale*[tiab] OR (multitrait[tiab] AND scaling[tiab] AND (analysis[tiab] OR analyses[tiab])) OR item discriminant[tiab] OR interscale correlation*[tiab] OR error[tiab] OR errors[tiab] OR "individual variability"[tiab] OR (variability[tiab] AND (analysis[tiab] OR values[tiab])) OR (uncertainty[tiab] AND (measurement[tiab] OR measuring[tiab])) OR "standard error of measurement"[tiab] OR sensitiv*[tiab] OR responsive*[tiab] OR ((minimal[tiab] OR minimally[tiab] OR clinical[tiab] OR clinically[tiab]) AND (important[tiab] OR significant[tiab] OR detectable[tiab]) AND (change[tiab] OR difference[tiab])) OR (small*[tiab] AND (real[tiab] OR detectable[tiab]) AND (change[tiab] OR difference[tiab])) OR meaningful change[tiab] OR "ceiling effect"[tiab] OR "floor effect"[tiab] OR "Item response model"[tiab] OR IRT[tiab] OR Rasch[tiab] OR "Differential item functioning"[tiab] OR DIF[tiab] OR "computer adaptive testing"[tiab] OR "item bank"[tiab] OR "cross-cultural equivalence"[tiab]) AND ("intensive care unit*" OR "critical care" OR ICU OR "critically ill" OR sepsis OR MODS OR "multi* organ dysfunction syndrome*" OR "intensive care" OR ITU OR "critical illness") AND (6MWT OR 6MWD OR 6MW OR "six minute walk* test*" OR "six-minute walk* test*" OR "6-minute walk* test*" OR "6 minute walk* test*" OR "six min*" OR "six-min*" OR "6 min*" OR 6-min* OR 6-MWT OR 6-MWD OR 6-MW OR "6 MWT" OR "6 MWD" OR "6 MW" OR "Barthel Index" OR "Barthel" OR "Lawton index" OR "IADL" OR "Katz" OR "ADL" OR "Katz ADL" OR "modified Katz" OR "Modified ADL" OR "sit-to-stand*" OR "sit to stand*" OR "chair-stand test*" OR "chair stand test*" OR "STS" OR "CST" OR "short physical performance battery" OR "SPPB" OR "hand-held dynamomet*" OR "handheld dynamomet*" OR "hand held dynamomet*" OR "hand dynamomet*" OR "hand-grip dynamomet*" OR "handgrip dynamomet*" OR "hand grip dynamomet*" OR "Jamar dynamomet*" OR "grip dynamomet*" OR "hand dynamomet*" OR hand-held dynamomet* OR handheld dynamomet* OR hand held dynamomet* OR HHD OR dynamomet* OR dynamomet* OR "hand strength*" OR "grip strength" OR "GLIM criteria" OR GLIM OR "Global Leadership Initiative on Malnutrition")

Supplementary Material

*Table S1. PRISMA Checklist*

| **Section and Topic** | **Item #** | **Checklist item** | **Location where item is reported** |
| --- | --- | --- | --- |
| **TITLE** | | |  |
| Title | 1 | Identify the report as a systematic review. | Page 1 |
| **ABSTRACT** | | |  |
| Abstract | 2 | See the PRISMA 2020 for Abstracts checklist. | Page 3 |
| **INTRODUCTION** | | |  |
| Rationale | 3 | Describe the rationale for the review in the context of existing knowledge. | Page 5 |
| Objectives | 4 | Provide an explicit statement of the objective(s) or question(s) the review addresses. | Page 6 Line 138 |
| **METHODS** | | |  |
| Eligibility criteria | 5 | Specify the inclusion and exclusion criteria for the review and how studies were grouped for the syntheses. | Page 7  Line 160 |
| Information sources | 6 | Specify all databases, registers, websites, organisations, reference lists and other sources searched or consulted to identify studies. Specify the date when each source was last searched or consulted. | Page 6  Line 149 |
| Search strategy | 7 | Present the full search strategies for all databases, registers and websites, including any filters and limits used. | Supplement Figure 1 |
| Selection process | 8 | Specify the methods used to decide whether a study met the inclusion criteria of the review, including how many reviewers screened each record and each report retrieved, whether they worked independently, and if applicable, details of automation tools used in the process. | Page 7  Line 168 |
| Data collection process | 9 | Specify the methods used to collect data from reports, including how many reviewers collected data from each report, whether they worked independently, any processes for obtaining or confirming data from study investigators, and if applicable, details of automation tools used in the process. | Page 7  Line 170 |
| Data items | 10a | List and define all outcomes for which data were sought. Specify whether all results that were compatible with each outcome domain in each study were sought (e.g. for all measures, time points, analyses), and if not, the methods used to decide which results to collect. | Page 7  Line 175 |
|  | 10b | List and define all other variables for which data were sought (e.g. participant and intervention characteristics, funding sources). Describe any assumptions made about any missing or unclear information. | Page 7  Line 171 |
| Study risk of bias assessment | 11 | Specify the methods used to assess risk of bias in the included studies, including details of the tool(s) used, how many reviewers assessed each study and whether they worked independently, and if applicable, details of automation tools used in the process. | Page 8  Line 190 |
| Effect measures | 12 | Specify for each outcome the effect measure(s) (e.g. risk ratio, mean difference) used in the synthesis or presentation of results. | Page 7  Line 175  Page 8  Line 204 |
| Synthesis methods | 13a | Describe the processes used to decide which studies were eligible for each synthesis (e.g. tabulating the study intervention characteristics and comparing against the planned groups for each synthesis (item #5)). | Page 8  Line 204 |
|  | 13b | Describe any methods required to prepare the data for presentation or synthesis, such as handling of missing summary statistics, or data conversions. | Page 8  Line 204 |
|  | 13c | Describe any methods used to tabulate or visually display results of individual studies and syntheses. | N/A |
|  | 13d | Describe any methods used to synthesize results and provide a rationale for the choice(s). If meta-analysis was performed, describe the model(s), method(s) to identify the presence and extent of statistical heterogeneity, and software package(s) used. | Page 8  Line 204 |
|  | 13e | Describe any methods used to explore possible causes of heterogeneity among study results (e.g. subgroup analysis, meta-regression). | Page 8  Line 209 |
|  | 13f | Describe any sensitivity analyses conducted to assess robustness of the synthesized results. | N/A |
| Reporting bias assessment | 14 | Describe any methods used to assess risk of bias due to missing results in a synthesis (arising from reporting biases). | N/A |
| Certainty assessment | 15 | Describe any methods used to assess certainty (or confidence) in the body of evidence for an outcome. | Page 8  Line 201 |
| **RESULTS** | | |  |
| Study selection | 16a | Describe the results of the search and selection process, from the number of records identified in the search to the number of studies included in the review, ideally using a flow diagram. | Page 9  Line 218 |
|  | 16b | Cite studies that might appear to meet the inclusion criteria, but which were excluded, and explain why they were excluded. | Figure 1 |
| Study characteristics | 17 | Cite each included study and present its characteristics. | Table 2 |
| Risk of bias in studies | 18 | Present assessments of risk of bias for each included study. | Page 9  Line 222  Supplement Table 6 |
| Results of individual studies | 19 | For all outcomes, present, for each study: (a) summary statistics for each group (where appropriate) and (b) an effect estimate and its precision (e.g. confidence/credible interval), ideally using structured tables or plots. | Supplement Table 4 |
| Results of syntheses | 20a | For each synthesis, briefly summarise the characteristics and risk of bias among contributing studies. | N/A |
|  | 20b | Present results of all statistical syntheses conducted. If meta-analysis was done, present for each the summary estimate and its precision (e.g. confidence/credible interval) and measures of statistical heterogeneity. If comparing groups, describe the direction of the effect. | Supplement Table 4 |
|  | 20c | Present results of all investigations of possible causes of heterogeneity among study results. | Supplement Table 5 |
|  | 20d | Present results of all sensitivity analyses conducted to assess the robustness of the synthesized results. | N/A |
| Reporting biases | 21 | Present assessments of risk of bias due to missing results (arising from reporting biases) for each synthesis assessed. | N/A |
| Certainty of evidence | 22 | Present assessments of certainty (or confidence) in the body of evidence for each outcome assessed. | Supplement Table 7 |
| **DISCUSSION** | | |  |
| Discussion | 23a | Provide a general interpretation of the results in the context of other evidence. | Page 14  Line 340 |
|  | 23b | Discuss any limitations of the evidence included in the review. | Page 16  Line 398 |
|  | 23c | Discuss any limitations of the review processes used. | Page 16  Line 398 |
|  | 23d | Discuss implications of the results for practice, policy, and future research. | Page 15  Line 377 |
| **OTHER INFORMATION** | | |  |
| Registration and protocol | 24a | Provide registration information for the review, including register name and registration number, or state that the review was not registered. | Page 6  Line 143 |
|  | 24b | Indicate where the review protocol can be accessed, or state that a protocol was not prepared. | Page 6  Line 143 |
|  | 24c | Describe and explain any amendments to information provided at registration or in the protocol. | N/A |
| Support | 25 | Describe sources of financial or non-financial support for the review, and the role of the funders or sponsors in the review. | Page 20  Line 495 |
| Competing interests | 26 | Declare any competing interests of review authors. | Page 19  Line 471 |
| Availability of data, code and other materials | 27 | Report which of the following are publicly available and where they can be found: template data collection forms; data extracted from included studies; data used for all analyses; analytic code; any other materials used in the review. | Page 19  Line 468 |

Supplementary Material

*Table S2. List of Comparator Instruments Used in Hypothesis Testing for Construct Validity*

| **Instrument** | **Construct** |
| --- | --- |
| Arm Muscle Area | Muscle mass |
| Berg Balance Scale (BBS) | Static balance |
| Center for epidemiologic studies-depression scale (CES-D) | Mental health |
| Dual-energy x-ray absorptiometry (DXA) | Body composition |
| Duke Activity Status Index | Functional capacity |
| Euro-QOL (EQ5D) – Mobility scale | Health related quality of life |
| Extra Short Musculoskeletal Function Assessment (XSFMA-F/B) | Functional status/disability |
| Fat Free Mass Index (FFMI) | Body composition |
| Forced expiratory volume (FEV_1_) | Respiratory function |
| Five Times Sit to Stand Test (FTSS) | Lower extremity strength |
| Functional Ambulation Category (FAC) | Functional mobility and gait |
| Functional Independence Measure (FIM) | Functional status/disability |
| Functional Performance Inventory (FPI) | Functional status/disability |
| Functional Status Score (FSS-ICU) | Physical function |
| Hospital Anxiety and Depression Scale (HADS) | Mental health |
| Handgrip strength (dynamometry) | Upper extremity strength |
| Handheld dynamometry – knee extension | Lower extremity strength |
| Impact of Event Scale-revised post-traumatic stress disorder (IES-R) | Mental health/PTSD |
| Kendall Muscle Testing - biceps | Upper extremity strength |
| Kendall Muscle Testing - quadriceps | Lower extremity strength |
| Maximum inspiratory pressure (MIP) | Respiratory muscle strength |
| Medical Research Council Manual Muscle Testing | Muscle strength |
| Patient- and Nutrition-Derived Outcome Risk Assessment score (PANDORA) | Nutritional status |
| Perceived Quality of Life Scale (PQOL) | Health related quality of life |
| Perme Intensive Care Unit Mobility Scale | Functional mobility |
| Phase angle | Body composition |
| Subjective Global Assessment Form (SGA) | Nutritional status |
| Short Form-12 Questionnaire (SF-32) – Mental health subscales | Mental health |
| Short Form-12 Questionnaire (SF-12) – Physical health subscales | Health related quality of life |
| Short Form-36 Questionnaire (SF-36) – Physical health subscales | Health related quality of life |
| Sickness impact profile | General health status |
| The Physical Function in ICU Test (PFIT-S) | Physical function |
| Timed Up and Go (TUG) | Mobility and balance |
| 4-metre timed walk | Gait speed |
| 6-minute walk test (6MWT) | Exercise capacity |

Supplementary Material

*Table S3. Hypotheses for Construct Validity and Responsiveness*

| **Specific hypotheses formulated for the CONCISE Measurement Instruments in relation to ‘Hypothesis Testing for Construct Validity’ and ‘Responsiveness’** | |
| --- | --- |
| **Hypothesis testing for construct validity** | Hypothesised a priori that we would observe:  **Comparison with other outcome measurement instruments (convergent validity)**  (1) In critically ill populations, large correlations ≥0.5 will be observed between CONCISE measurement instruments and comparator instruments (Table S2) measuring similar constructs.  (2) In critically ill populations, moderate to large correlations (≥0.3 and ≤0.7) will be observed between CONCISE measurement instruments and instruments (Table S2) measuring related but dissimilar constructs (e.g., muscle strength for a measure of physical function)  **Comparison with other outcome measurement instruments (divergent validity)**  (3) In critically ill populations, small correlations (≤0.3) will be observed between CONCISE measurement instruments and instruments (Table S2) measuring unrelated constructs (e.g., mental health for a measure of physical function)  **Comparison between subgroups (Divergent validity)**  (4) CONCISE measurement instrument scores should be able to distinguish between those who score high or low on measures of similar constructs, with effect sizes of ≥0.5 OR AUC ≥ 0.7.  (5) CONCISE measurement instrument scores should be able to distinguish between the following groups, with effect sizes of ≥0.2 OR AUC ≥ 0.7:  a) younger vs. older age groups, with older groups having worse physical and muscle function and reduced ADL score  b) comorbid vs not comorbid, with more comorbid groups having worse physical and muscle function and reduced ADL score |
| **Responsiveness** | Hypothesised *a priori* that we would observe:   \| ***Before and After Intervention/Recovery over time***  (6) In response to an intervention, we expect a moderate to large improvement in a CONCISE measurement instrument (i.e., effect sizes ≥0.5) in the intervention group, but not the control group.  (7) In response to an intervention, we expect a moderate to large difference in post-intervention CONCISE measurement instrument scores (or change scores) between the intervention and control groups, with the intervention group showing greater improvement (i.e., effect sizes ≥0.5).    ***Comparison between subgroups***  (8) A medium to large difference (i.e., effect sizes ≥0.5) in CONCISE measurement instrument change scores between groups with different levels of impairment (e.g., ADL score, HRQOL score, self-reported scores).  (9) The CONCISE measurement instruments can discriminate between improved and unimproved groups (i.e., AUC ≥ 0.70) following the intervention or over time.  ***Comparison with other outcome measurement instruments***  (10) Changes in CONCISE measurement instrument scores will have moderate to large correlations (i.e. ≥0.30) with instruments measuring similar constructs (Table S2) \| \| --- \| |

ADL = activities of daily living; AUC = area under the curve; HRQOL = health related quality of life

Supplementary Material

*Table S4. COSMIN Risk of Bias Ratings*

| **Study** | **4.Internal Consistency** | **6.Reliability** | **7.Measurement Error** | **9.Hypotheses Testing for Construct Validity** | **10.Responsiveness** |
| --- | --- | --- | --- | --- | --- |
| Abd-El-Gawad 2013 (1) | NA | NA | NA | NA | NA |
| Ali 2008 (2) | NA | NA | NA | Adequate | NA |
| Alison 2012 (3) | NA | NA | NA | Very Good | Very Good |
| Bakhru 2018 (4) | NA | NA | NA | NA | NA |
| Baldwin 2013 (5) | NA | Doubtful | Doubtful | NA | NA |
| Bo 2003 (6) | NA | NA | NA | NA | NA |
| Broslawski 1995 (7) | NA | NA | NA | NA | NA |
| Bruno 2022 (8) | NA | NA | NA | NA | NA |
| Chan 2015 (9) | NA | NA | Very Good | Very Good | Very Good |
| Chan 2016 (10) | NA | Doubtful | Doubtful | Very Good | Doubtful |
| Chan 2017 (11) | NA | NA | NA | Very Good | NA |
| Chan 2018 (12) | NA | NA | NA | Very Good | NA |
| Chiang 2006 (13) | NA | NA | NA | NA | Doubtful |
| Chrispin 1997 (14) | Very Good | NA | NA | NA | NA |
| Clini 2011 (15) | NA | NA | NA | Doubtful | Doubtful |
| Costigan 2019 (16) | NA | Inadequate | Inadequate | NA | NA |
| Cottereau 2015 (17) | NA | NA | NA | NA | NA |
| Daubin 2011 (18) | NA | NA | NA | NA | NA |
| de Azevedo 2021 (19) | NA | NA | NA | NA | Doubtful |
| Denehy 2014 (20) | NA | NA | NA | Adequate | NA |
| dos Reis 2022 (21) | Doubtful | Doubtful | Doubtful | Doubtful | NA |
| Fan 2014 (22) | NA | NA | NA | Doubtful | NA |
| Hermans 2012 (23) | NA | Inadequate | NA | NA | NA |
| Heyland 2000 (24) | Doubtful | Doubtful | NA | Doubtful | NA |
| Kaarola 2004 (25) | NA | NA | NA | NA | NA |
| Kawakami 2021 (26) | NA | NA | Inadequate | NA | Doubtful |
| Khoudri 2007 (27) | Very Good | Adequate | NA | Adequate | NA |
| Lee et al, 2012 (28) | NA | NA | NA | Doubtful | NA |
| Melo 2019 (29) | NA | Doubtful | Doubtful | NA | NA |
| Melo 2022 (30) | NA | Adequate | NA | Very Good | Doubtful |
| Mohamed-Hussein 2017 (31) | NA | NA | NA | NA | NA |
| Needham 2014 (32) | NA | NA | NA | Very Good | NA |
| O'Grady 2022 (33) | NA | NA | Doubtful | NA | NA |
| Parry 2015 (34) | NA | NA | Doubtful | Very Good | Doubtful |
| Parry 2021 (35) | NA | NA | NA | NA | Very Good |
| Parry, Berney 2015 (36) | NA | Doubtful | NA | Doubtful | NA |
| Puthucheary 2020 (37) | NA | NA | NA | Very Good | Doubtful |
| Rosa 2020 (38) | NA | NA | NA | Doubtful | NA |
| Sacanella 2009 (39) | NA | NA | NA | NA | NA |
| Shahbazi 2021 (40) | NA | NA | NA | Very Good | NA |
| Theilla 2021 (41) | NA | NA | NA | Very Good | NA |
| Tripathy 2014 (42) | NA | NA | NA | NA | NA |
| Van Der Schaff 2008 (43) | NA | NA | NA | Very Good | NA |
| Vest 2011 (44) | NA | NA | NA | Doubtful | NA |
| Weinert 1997 (45) | NA | NA | NA | Inadequate | NA |
| Wischmeyer 2017 (46) | NA | NA | NA | NA | Doubtful |
| Wu 1995 (47) | NA | NA | NA | Very Good | NA |

NA = not applicable

Supplementary material

*Table S5. Grading of Recommendations, Assessment, Development and Evaluation system approach (GRADE) ratings*

|  | **Risk of Bias** | **Inconsistency** | **Imprecision** | **Indirectness** | **Overall** |
| --- | --- | --- | --- | --- | --- |
| **Internal consistency** | | | | | |
| SF-36 PF | No ROB | No inconsistency | No imprecision | No indirectness | High |
| SF-36 PCS | NA | NA | NA | NA | NA |
| STS | NA | NA | NA | NA | NA |
| 6MWT | NA | NA | NA | NA | NA |
| SPPB | NA | NA | NA | NA | NA |
| SPPB - 4m gait speed | NA | NA | NA | NA | NA |
| Barthel Index | Very serious (-2) | No inconsistency | No imprecision | No indirectness | Low |
| Katz Index | NA | NA | NA | NA | NA |
| IADL | NA | NA | NA | NA | NA |
| Handgrip strength | NA | NA | NA | NA | NA |
| GLIM | NA | NA | NA | NA | NA |
| **Reliability** | | | | | |
| *Test-retest* | | | | | |
| SF-36 PF | Serious (-1) | Serious (-1) | No imprecision | No indirectness | Low |
| SF-36 PCS | NA | NA | NA | NA | NA |
| STS | Very serious (-2) | No inconsistency | Serious imprecision (-1) | No indirectness | Very low |
| 6MWT | NA | NA | NA | NA | NA |
| SPPB | NA | NA | NA | NA | NA |
| SPPB - 4m gait speed | Very serious (-2) | No inconsistency | No imprecision | No indirectness | Low |
|  |  |  |  |  |  |
| Barthel Index | NA | NA | NA | NA | NA |
| Katz Index | NA | NA | NA | NA | NA |
| IADL | NA | NA | NA | NA | NA |
| Handgrip strength | Very serious (-2) | No inconsistency | Very serious imprecision (-2) | No indirectness | Very low |
| GLIM | NA | NA | NA | NA | NA |
| *Inter-rater Reliability* |  |  |  |  |  |
| SF-36 PF | NA | NA | NA | NA | NA |
| SF-36 PCS | NA | NA | NA | NA | NA |
| STS | Serious (-1) | No inconsistency | No imprecision | No indirectness | Moderate |
| 6MWT | NA | NA | NA | NA | NA |
| SPPB | NA | Na | NA | NA | NA |
| SPPB - 4m gait speed | Very serious (-2) | No inconsistency | No imprecision | No indirectness | Low |
| Barthel Index | Very serious (-2) | No inconsistency | No imprecision | No indirectness | Low |
| Katz Index | NA | NA | NA | NA | NA |
| IADL | NA | NA | NA | NA | NA |
| Handgrip strength | Serious (-1) | No inconsistency | Serious (-1) | No indirectness | Low |
| GLIM | NA | NA | NA | NA | NA |
| **Measurement error** | | | | | |
| SF-36 PF | NA | NA | NA | NA | NA |
| SF-36 PCS | Extremely serious (-3) | No inconsistency | Serious (-1) | No indirectness | Very Low |
| STS | Serious (-1) | Serious (-1) | No imprecision | No indirectness | Low |
| 6MWT | No ROB | No inconsistency | No imprecision | No indirectness | High |
| SPPB | Very serious (-2) | No inconsistency | Very serious imprecision (-2) | No indirectness | Very low |
| SPPB - 4m gait speed | Very serious (-2) | No inconsistency | No imprecision | No indirectness | Low |
| Barthel Index | Very serious (-2) | No inconsistency | No imprecision | No indirectness | Low |
| Katz Index | NA | NA | NA | NA | NA |
| IADL | NA | NA | NA | NA | NA |
| Handgrip strength | Very serious (-2) | No inconsistency | Very serious imprecision (-2) | No indirectness | Very low |
| GLIM | NA | NA | NA | NA | NA |
| **Hypotheses testing for Construct Validity** | |  | |  | |
| SF-36 PF | No ROB | No inconsistency | No imprecision | No indirectness | High |
| SF-36 PCS | No ROB | No inconsistency | No imprecision | No indirectness | High |
| STS | No ROB | No inconsistency | No imprecision | No indirectness | High |
| 6MWT | No ROB | No inconsistency | No imprecision | No indirectness | High |
| SPPB | No ROB | No inconsistency | Serious (-1) | Serious (-1) | Low |
| SPPB - 4m gait speed | No ROB | No inconsistency | No imprecision | No indirectness | High |
|  |  |  |  |  |  |
| Barthel Index | No ROB | No inconsistency | No imprecision | No indirectness | High |
| Katz Index | No ROB | No inconsistency | No imprecision | No indirectness | High |
| IADL | No ROB | No inconsistency | Serious (-1) | No indirectness | Moderate |
| Handgrip strength | No ROB | Serious (-1) | No imprecision | No indirectness | Moderate |
| GLIM | No ROB | No inconsistency | No imprecision | No indirectness | High |
| **Responsiveness** | | | | | |
| SF-36 PF | Serious (-1) | No inconsistency | No imprecision | No indirectness | Moderate |
| SF-36 PCS | Serious (-1) | No inconsistency | No imprecision | No indirectness | Moderate |
| STS | Very serious (-2) | No inconsistency | No imprecision | No indirectness | Low |
| 6MWT | No ROB | No inconsistency | No imprecision | No indirectness | High |
| SPPB | Very serious (-2) | No inconsistency | Serious imprecision (-1) | No indirectness | Very low |
| SPPB - 4m gait speed | NA | NA | NA | NA | NA |
| Barthel Index | Very serious (-2) | No inconsistency | Very serious (-2) | No indirectness | Very low |
| Katz Index | Very serious (-2) | No inconsistency | Serious (-1) | No indirectness | Very low |
| IADL | NA | NA | NA | NA | NA |
| Handgrip strength | NA | NA | NA | NA | NA |
| GLIM | NA | NA | NA | NA | NA |

GLIM = global leadership initiative on malnutrition; IADL = instrumental activities of daily living; NA = not applicable; ROB = risk of bias; SF-36 = Short Form-36 Questionnaire; SPPB = short physical performance battery; STS = sit-to-stand; 6MWT = 6-minute walk test

Supplementary material

*Table S6. Results of Individual Studies*

| **Reliability** | | | | | | | | | | | | |
| --- | --- | --- | --- | --- | --- | --- | --- | --- | --- | --- | --- | --- |
|  | Internal consistency  (Cronbach α) | | | Reliability Test-retest  (ICC) | | | Reliability Inter-Rater  (ICC) | | | Measurement Error | | |
|  | n | Meth qual | Result (rating) | n | Meth qual | Result (rating) | n | Meth qual | Result (rating) | n | Meth qual | Result (rating) |
| **SF-36 PF** | | | | | | | | | | | | |
| Chrispin 1997 (14) | 166 | V | 0.93  (?) | - | - | - | - | - | - | - | - | - |
| Heyland 2000 (24) | 30 | D | 0.94  (?) | 30 | D | 0.56  (-) | - | - | - | - | - | - |
| Khoudri 2007 (27) | 145 | V | 0.96  (?) | 73 | A | 0.98  (+) |  |  |  |  |  |  |
| **Pooled or summary result**  **(overall rating)** | 3 studies  n = 341 | 2 = V  1 = D | Range 0.93–0.96  Weighted mean (SD)  0.94 (0.01)  (?)  Indeterminate results due to no evidence for structural validity | 2 studies  n = 103 | 1 = A  1 = D | Range  0.56-0.98  Weighted mean (SD)  0.86 (0.19)  (+) |  |  |  |  |  |  |
| **SF-36 PCS** | | | | | | | | | | | | |
| Kawakami 2021 (26) | - | - | - | - | - | - | - | - | - | 96 | I | SF36 PCS  MIC = 6.5 points  (?) |
| **STS** | | | | | | | | | | | | |
| Costigan 2019 (16) | - | - | - | - | - | - | 35 | I | 0.85  (+) | 35 | I | SEM 1.91  repetitions  SDC90 4.45 repetitions  (?) |
| Melo 2019  (5xSTS)  (29) | - | - | - | 96 | D | 0.99  (+) | 96 | D | 0.99  (+) | 96 | D | SEM 0.68 and 0.69 seconds  (5xSTS) |
| Melo 2022  (5xSTS)  (30) | - | - | - | - | - | - | 142 | A | 0.92  (+) | - | - | - |
| O’Grady 2022 (33) | - | - | - | - | - | - | - | - | - | 80 | D | SEM 0.51 repetitions  SDC90 1.19 repetitions  (?) |
| **Pooled or summary result**  **(overall rating)** |  |  |  | 1 study  n = 96 | 1 = D | 0.99  (+) | 3 studies  n = 273 | 1 = A  1 = D  1 = I | Range 0.85-0.99  Pooled ICC (95% CI)  0.95 (0.77-0.99)  (+)  (I^2^=98%) | 3 studies  n = 211 | 2 = D  1 = I | SEM  0.51-1.91  (30STS)  0.68-0.69s (FTSTT)  SDC90 1.19-4.45  (30STS)  (?) |
| **6MWT** | | | | | | | | | | | | |
| Chan 2015 (9) | - | - | - | - | - | - | - | - | - | 651 | V | SEM  31-38m  SDC 0.2 SD  (small effect size)  21-34m  SDC90  67-88m  MIC (anchor based)  14-30m  (-) |
| **SPPB** | | | | | | | | | | | | |
| Parry 2015 (34) | - | - | - | - | - | - | - | - | - | 23 | D | MIC 1.3 points  (?) |
| **SPPB – 4m gait speed** | | | | | | | | | | | | |
| Chan 2016 (10) |  |  |  | 306 | D | ICC Range 0.89-0.99  (+)  Pooling not possible due to lack of data | 306 | D | Pooled  ICC  0.97  (+) | 306 | D | SEM  0.06m/s  SDC 0.2SD  (small effect size)  0.06m/s  SDC90  0.13-0.14m/s  MIC (anchor based)  0.03-0.04m/s  (-) |
| **Activities of Daily Living - Barthel Index** | | | | | | | | | | | | |
| dos Reis 2022 (21) | 122 | D | 0.76-0.82  Total score 0.81  (?)  Indeterminate results due to no evidence for structural validity | - | - | - | 122 | D | 0.98  (+) | 122 | D | SDC 20 points  (?) |
| **Activities of Daily Living - Katz Index** | | | | | | | | | | | | |
| No studies | - | - | - | - | - | - | - | - | - | - | - | - |
| **Activities of Daily Living - IADL** | | | | | | | | | | | | |
| No studies | - | - | - | - | - | - | - | - | - | - | - | - |
| **Handgrip Strength** | | | | | | | | | | | | |
| Baldwin 2013 (5) | - | - | - | 17 | D | RHS 0.92  LHS 0.86  (+) | 17 | D | RHS 0.92  LHS 0.89  (+) | 17 | D | RHS  SEM 2.8kg  SDC95 7.8kg  LHS  SEM 4.5kg  SDC95 12.5kg  (?) |
| Hermans 2012 (23) | - | - | - | - | - | - | 46 | I | RHS 0.93  LHS 0.97  (+) | - | - | - |
| Parry, Berney 2015 (36) |  |  |  |  |  |  | 29 | D | RHS 0.93  LHS 0.98  (+) |  |  |  |
| **Pooled or summary result**  **(overall rating)** | - | - | - | 1 study  n = 17 | 1 = D | Range 0.86-0.92  (+)  Mean (SD) 0.89 (0.03)  (+) | 3 studies  n = 92 | 2 = D  1 = I | Range 0.89-0.98  Pooled ICC (95% CI)  0.95  (0.91-0.97)  (+)  (I^2^=64%) | 1 study  n = 17 | 1 = D | SEM Range 2.8-4.5kg  SDC95 Range  7.8-12.5kg  (?) |
| **GLIM** | | | | | | | | | | | | |
| No studies | - | - | - | - | - | - | - | - | - | - | - | - |
| **Validity and Responsiveness** | | | | | | | | | | | | |
|  | Predictive Validity | | | | Hypothesis testing for construct validity | | | | Responsiveness | | | |
|  | n | Meth qual | Results | | n | Meth qual | Results (rating) | | n | Meth qual | Results (rating) | |
| **SF-36 PF** | | | | | | | | | | | | |
| Alison 2012 (3) | - | - | - | | 162  140  128 | V | 6MWT Wk 1  r = 0.62 (+)  6MWT Wk 8  r = 0.55 (+)  6MWT Wk 26  r = 0.47 (-) | | - | - | - | |
| Bakhru 2018 (4) | 30 | - | Measured at 1 month post discharge  1-year mortality  OR 0.94 (95% CI 0.88-1.01), p = 0.11  6-month readmissions  OR 0.97 (95% CI 0.93-1.01), p = 0.18 | | - | - | - | | - | - | - | |
| Chan 2015 (9) | - | - | - | | 1461-1470 | V | 6MWT  Weighted Mean (SD) r = 0.62 (0.11) (+) | | - | - | - | |
| Chan 2018 (12) | - | - | *-* | | 86  84 | V | DXA lean body mass at 6 months  r = 0.28 (-)  DXA lean body mass at 12 months  r = 0.07 (-) | | - | - | - | |
| Denehy 2014 (20) | - | - | *-* | | 123  126 | A | 6MWT  r = 0.69 (+)  TUG  r = -0.68 (+) | | - | - | - | |
| Khoudri 2007 (27) | - | - | - | | 145  145 | A | Age in years  (<23, 23-33, 34-52, >53)  ES = 0.62 (+)  Charlson Comorbidity Index (0, 1, >2)  ES = 0.47 (+) | | - | - | - | |
| Needham 2014 (32) | - | - | - | | 153  165  164  156  157  154 | V | Arm muscle area  r = -0.04 (-)  Muscle strength  r = 0.44 (+)  Handgrip strength  r = 0.32 (+)  MIP  r = 0.35 (+)  4m gait speed  r = 0.56 (+)  6MWT  r = 0.54 (+) | | - | - | - | |
| Puthucheary 2020 (37) | - | - | - | | 159  159  159 | V | XSFMA-F  r = -0.89 (+)  XSFMA-B  r = -0.82 (+)  ADL  r = -0.73 (+) | | 159 | D | **Comparison of subgroups**  Complete recovery group post-ICU discharge  6 months ES 1.75 (+)  12 months ES 2.05 (+)  24 months ES 1.63 (+)  Persistent impairment group post-ICU discharge  6 months ES 0.71  12 months ES 0.42  24 months ES 0.37 | |
| Wischmeyer 2017 (46) |  |  |  | |  |  |  | | 54 | D | **Before and after intervention/Recovery over time**  Control group  3 months vs 6 months  ES 0.00 (-0.51 - 0.50)  Intervention group  3 months vs 6 months  ES 0.47 (-0.13 - 1.07)  (-) | |
| **Pooled or summary result**  **(overall rating)** |  | 1 study  n = 30 | Not predictive of 1-year mortality or 6-month readmissions | | 7 studies  n = 2253 - 2313 | 4 = V  3 = A | Majority of results (15/19, 79%) supported our hypotheses (+)  6 studies investigated convergent validity. Majority (13/17, 76%) supported our hypotheses  1 study investigated known groups validity. All results (2/2, 100%) supported our hypotheses | | 2 studies  n = 213 | 1 = A  1 = D | Majority of our results (3/4, 75%) supported our hypotheses (+)  1 study involved the difference in SF-36 PF over time in different subgroups and all results (3/3, 100%) supported our hypotheses  1 study involved measuring the SF-36 over time following an intervention and did not support our hypotheses | |
| **SF-36 PCS** | | | | | | | | | | | | |
| Bakhru 2018 (4) | 30 | - | Measured at 1 month post ICU discharge  1-year mortality  OR 0.95 (95% CI 0.89-1.01), p = 0.12  6-month readmissions  OR 0.98 (95% CI 0.94-1.02), p = 0.36 | | - | - | - | | - | - | - | |
| Chan 2017 (11) | - | - | - | | 233  233  233  233  134  233  99  99  134  233 | V | Arm muscle area  r = 0.16 (-)  Muscle strength (MRC)  r = 0.32 (+)  Handgrip strength  r = 0.19 (-)  MIP  r = 0.20 (+)  4m gait speed  r = 0.46 (-)  6MWT  r = 0.43 (-)  IADL  r = -0.46 (+)  Katz Index  r = -0.06 (-)  FPI  r = 0.59 (+)  FEV_1_  r = 0.19 (+) | | - | - | - | |
| de Azevedo 2021 (19) |  |  |  | |  |  |  | | 181 | D | **Recovery over time after intervention**  Intervention group  Median difference (IQR) 9.23 (0.00 – 22.49)  Control group  Median difference (IQR)  0.00 (0.00 – 18.10)  (?) | |
| Denehy 2014 (20) | - | - | - | | 122  125 | A | 6MWT  r = 0.50 (+)  TUG  r = -0.54 (+) | | - | - | - | |
| Heyland 2000 (24) | - | - | - | | 30 | D | PQOL   1. r = 0.45 (+) | | - | - | - | |
| Kawakami 2021 (26) | - | - | - | | - | - | - | | 96 | D | **Comparison with other outcome measures**  Global Assessment Rating negative change and change in SF-36 PCS  ES 0.72  (+) | |
| Puthucheary 2020 (37) | - | - | - | | 159  159  159 | V | XSFMA-F  r = -0.80 (+)  XSFMA-B  r = -0.75 (+)  ADL  r = -0.61 (+) | | 159 | D | **Comparison between subgroups**  Complete recovery group post-ICU discharge  6 months ES 1.00 (+)  12 months ES 1.44 (+)  24 months ES 1.14 (+)  Persistent impairment group post-ICU discharge  6 months ES 0.01  12 months ES 0.25  24 months ES 0.15 | |
| Weinert 1997 (45) | 24 | - | - | | 24  24  24 | I | Life satisfaction  r = -0.32 (+)  Health satisfaction  r = -0.72 (+)  Karnofsky index  r = -0.75 (+)  SF-36 MCS  r = 0.10 (+)  CES-D  r = -0.17 (+) | | - | - | **-** | |
| Wischmeyer 2017 (46) |  |  |  | |  |  |  | | 42 | D | **Before and after intervention/Recovery over time**  Control group  3 months vs 6 months  ES 0.05  (95%CI -0.48 - 0.57)  Intervention group  3 months vs 6 months  ES 0.59  (95%CI -0.03 - 1.21)  (+) | |
| **Pooled or summary result**  **(overall rating)** | 2 studies  n = 54 | - | Not predictive of 1-year mortality, 6-month readmissions | | 5 studies  n = 434 -571 | 2 = V  1 = A  1 = D  1 = I | Majority of results (16/21, 76%) supported our hypotheses (+)  5 studies investigated convergent validity. 72% (13/18) of results supported our hypotheses  2 studies investigated divergent validity. All results (4/4, 100%) supported our hypotheses | | 4 studies  n = 478 | 4 = D | Majority of our results (5/6, 83%) supported our hypotheses (+)  1 study involved the comparison of the SF-36 PCS to another outcome measurement instrument and supported our hypotheses  1 study involved the difference in SF-36 PCS over time in different subgroups and all results (3/3, 100%) supported our hypotheses  1 study involved measuring the SF-36 over time following an intervention, medians were reported so effect sizes were unable to be calculated | |
| **STS** | | | | | | | | | | | | |
| Denehy 2014 (20) | - | - | - | | 26  26 | A | 6MWT  r = -0.69 (+)  TUG  r = 0.82 (+) | | - | - | - | |
| Melo 2022 (30) | 142 | - | Measured at ICU discharge  Hospital LOS r = -0.56  ICU LOS r = -0.49  Duration of MV r = -0.27 | | 142  142 | V | FIM  r = -0.67 (+)  Handgrip strength  r = -0.57 (+) | | 142 | D | **Comparison between subgroups**  (5xSTS)  A threshold of 23.5 seconds was linked to ICU readmission  AUC 0.94 (+) | |
| **Pooled or summary result**  **(overall rating)** | 1 study  n = 142 | - | Predictive of hospital length of stay | | 2 studies  n = 319 | 1 = V  1 = A | Majority of results (4/4, 100%) supported our hypotheses (+)  2 studies investigated convergent validity. All results (4/4, 100%) supported our hypotheses | | 1 study  n = 142 | 1 = D | 1 study involved the comparison of subgroups for STS and supported our hypothesis (+) | |
| **6MWT** | | | | | | | | | | | | |
| Alison 2012 (3) | - | - | - | | 162  140  128 | V | SF-36 PF Wk 1  r = 0.62 (+)  SF-36 PF Wk 8  r = 0.55 (+)  SF-36 PF Wk 26  r = 0.47 (-) | | 173 | V | **Recovery over time**  Change in 6MWT from Wk1 to Wk8 ES 0.76 (95%CI 0.53-0.99) (+)  Wk1 to Wk26 ES 0.93  (95% CI 0.69-1.17) (+) | |
| Chan 2015 (9) | 237  226  143  287  297  294 | - | Measured at  3 months  1-year mortality OR 0.86 (95% CI 0.80-0.94), p<0.01  Hospitalisation between 6-12months  OR 0.91 (95% CI 0.85-0.98), p<0.01  Return to normal activity  OR 1.09 (95% CI 1.01-1.18), p<0.05  Measured at  6 months  1-year mortality OR 0.84 (95% CI 0.77-0.91), p<0.01  Hospitalisation between 6-12months  OR 0.91 (95% CI 0.86-0.96), p<0.01 (n=326)  Return to normal activity  OR 1.12 (95% CI 1.05-1.20), p<0.01 (n=111) | | 1461-1470  297-304  643-652  297-304  1461-1470  643-652  643-652  643-652 | V | SF -36 PF  Weighted Mean (SD)  r = 0.62 (0.11) (+)  FPI  Weighted Mean (SD)  r = 0.55 (0.04) (+)  EQ5D Mobility  Weighted Mean (SD)  r = 0.56 (0.04) (+)  4m gait speed  Weighted mean (SD)  r = 0.63 (0.04) (+)  SF-36 MH domain  Weighted Mean (SD)  r = 0.22 (0.08)  (+)  EQ-5D Anxiety  Weighted mean (SD)  r = -0.17 (0.04)  (+)  HADS Anxiety Symptoms  Weighted mean (SD)  r = -0.12 (0.05)  (+)  IES-R PTSD  Weighted mean (SD)  r = -0.16 (0.08)  (+) | | 651 | V | **Comparison between subgroups**  Change in 6MWT discriminated between  the substantial vs modest or no improvement groups  6 months AUC 0.79 (95% CI 0.67-0.90) (+)  12 months AUC 0.79 (95% CI 0.66-0.92) (+) | |
| Chan 2017 (11) | 224 | - | Measured at 6 months  No hospitalisation between 6-12months  OR 1.27 (95% CI 1.08 to 1.50), p <0.01 | | 233  233 | V | SF-36 PCS  r = 0.43 (-)  EQ-5D utility  r = 0.34 (+) | | - | - | **-** | |
| Chan 2018 (12) | - | - | - | | 85  84 | V | DXA lean body mass at 6 months  r = 0.42 (+)  DXA lean body mass at 12 months  r = 0.36 (+) | | - | - | **-** | |
| Denehy 2014 (20) | - | - | - | | 26  127  123  122  26 | A | STS  r = -0.69 (+)  TUG  r = -0.79 (+)  SF-36 PF  r = 0.69 (+)  SF-36 PCS  r = -0.54 (+)  Berg balance scale  r = -0.80 (+) | | - | - | - | |
| Needham 2014 (32) | - | - | - | | 148  156  155  148  153  154 | V | Arm muscle area  r = -0.06 (-)  Muscle strength  r = 0.32 (+)  Handgrip strength  r = 0.28 (-)  MIP  r = 0.40 (-)  4m timed walk  r = 0.52 (+)  SF-36 PF  r = 0.54 (+) | | - | - | - | |
| Rosa 2020 (38) | - | - | - | | 32 | D | Prediction of physical function improvement as measured by Barthel Index  AUC 0.72 (95% CI 0.53-0.88) (+) | | - | - | **-** | |
| Parry 2021 (35) | - | - | - | | - | - | - | | 764 | V | **Recovery over time**  Change in 6MWT from Month 3 to Month 12  Mean difference  ES 0.13  (95%CI 0.03-0.24) (-) | |
| **Pooled or summary result**  **(overall rating)** | 2 studies  n = 875 | - | Predictive of 1-year mortality, hospital readmissions and return to normal activity. | | 7 studies  n = 948 - 2265 | 4 = V  2 = A  1 = D | Majority of results (22/26, 85%) supported our hypotheses (+)  7 studies investigated convergent validity. Majority of results (18/21, 86%) supported our hypotheses  2 studies investigated divergent validity. Majority of results (4/5, 80%) supported our hypotheses | | 3 studies  n = 1588 | 3 = V | Majority (4/5, 80%) of results support our hypotheses (+)  2 studies involved the comparison of the 6MWT before and after intervention. 67% (2/3) of results supported our hypotheses  1 study involved the comparison of subgroups for the 6MWT. All results (2/2, 100%) supported our hypotheses | |
| **SPPB** | | | | | | | | | | | | |
| Bakhru 2018 (4) | 35 | - | 1-year mortality OR 0.73 (95% CI 0.52-1.04); p = 0.05  6-month readmission  OR 0.83 (95% CI 0.66-1.05); p = 0.11 | | - | - | - | | - | - | - | |
| Parry 2015 (34) | - | - | - | | 66 | V | Muscle strength (MRC)  r = 0.30 (+) | | 66 | D | **Before and after intervention/Recovery over time**  Change in SPPB from awakening to ICU discharge ES 0.33 (-) | |
| **Pooled or summary result**  **(overall rating)** | 1 study  n = 36 | - | Not predictive of 1-year mortality or 6-month readmission | | 1 study  n = 66 | 1 = V | 1 study investigated convergent validity. The results (1/1, 100%) supported our hypotheses (+) | | 1 study  n = 66 | 1 = D | 1 study involved the comparison of the SPPB before and after intervention and did not support our hypotheses  (-) | |
| **SPPB – 4m gait speed** | | | | | | | | | | | | |
| Chan 2015 (9) | - | - | - | | 297-304 | V | 6MWT  Weighted mean (SD) r = 0.63 (0.04) (+) | | - | - | - | |
| Chan 2017 (11) | 129 | - | No hospitalisation between 6-12months  OR 1.29 (95% CI 1.06 to 1.58), p <0.05 | | 134  134 | V | SF-36 PCS  r = 0.46 (-)  EQ-5D Utility  r = 0.44 (+) | | - | - | - | |
| Chan 2016 (10) | - | - | - | | 296-310  293-313  293-313  620-623  620-623  620-623  620-623 | V | 6MWT, SF-36 and EQ-5D data included above in Chan 2015 and 2016  FPI  r = 0.5 (+)  ADL dependencies  r = -0.27 (-)  IADL dependencies  r = -0.43 (+)  SF-36 MCS  Weighted Mean (SD)  r = 0.23 (0.03)  (+)  EQ-5D Anxiety  Weighted mean (SD)  r = -0.19 (0.01)  (+)  HADS Anxiety Symptoms  Weighted mean (SD)  r = -0.21 (0.04)  (+)  IES-R PTSD  Weighted mean (SD)  r = -0.26 (0.03)  (+) | | 203 | D | **Comparison between subgroups**  Statistically significant increases in gait speed were observed for patients with a more than 10-point increase in the SF-36 PF domain. Unable to calculate effect sizes (?) | |
| Chan 2018 (12) | - | - | - | | 88  84 | V | DXA lean body mass at 6 months  r = 0.41 (+)  DXA lean body mass at 12 months  r = 0.27 (-) | | - | - | - | |
| Needham 2014 (32) | - | - | - | | 157  153  161 | V | SF-36 PF  r = 0.56 (+)  6MWT  r = 0.52 (+)  Muscle Strength  r = 0.38 (+) | | - | - | - | |
| **Pooled or summary result**  **(overall rating)** | 1 study  n = 129 | - | Predictive of hospital readmission between 6-12months | | 5 studies  n = 961 - 1310 | 4 = V  1 = A | Majority of results (12/15, 80%) supported our hypotheses (+)  5 studies investigated convergent validity. 73% (8/11) of results supported our hypotheses  1 study investigated divergent validity. All results (4/4, 100%) supported our hypotheses | | 1 study  n = 203 | 1 = D | 1 study involved the comparison of the 4m gait speed test between subgroups with inconclusive results (?) | |
| **Activities of Daily Living - Barthel Index** | | | | | | | | | | | | |
| Chiang 2006 (13) | - | - | - | | - | - | - | | 32 | D | **Before and after intervention**  Change in Barthel Index after:  3 weeks ES 1.03  (95%CI 0.27-1.74) (+)  6 weeks ES 2.02  (95%CI 1.12-2.81) (+) | |
| Dos Reis 2022 (21) | - | - | - | | 122  122  122  122  122  122 | D | Perme ICU Mobility  r = 0.85 (+)  FSS-ICU  r = 0.88 (+)  PFIT-s  r = 0.86 (+)  MRC-ss  r = 0.65 (+)  Handgrip strength  r = 0.57 (+)  HHD knee extension  r = 0.62 (+) | | - | - | - | |
| Sacanella 2009 (39) | 230 | - | Univariate analysis  In hospital mortality  p = 0.15  Post-hospital mortality  p = 0.09  Cumulative mortality  p = 0.03 | | - | - | - | | - | - | - | |
| Van Der Schaaf 2008 (43) | 69 | - | Duration of MV  r = -0.40; p < 0.01 | | 69  69 | V | FAC  r = 0.76 (+)  Handgrip strength  r = 0.62 (+) | | - | - | - | |
| **Pooled or summary result**  **(overall rating)** | 2 studies  n = 299 | - | Conflicting results for mortality. Predictive of the duration of mechanical ventilation | | 2 studies  n = 191 | 1 = V  1 = D | Majority of results (8/8, 100%) supported our hypotheses (+)  2 studies investigated convergent validity. All results (8/8, 100%) supported our hypotheses | | 1 study  n = 32 | 1 = D | 1 study involved the comparison of the Barthel Index before and after intervention and supported our hypotheses (+) | |
| **Activities of Daily Living - Katz Index** | | | | | | | | | | | | |
| Abd-El-Gawad 2013  (1) | 65 | - | 30-day mortality  AUC 0.71  Duration of MV  AUC 0.68 | | - | - | - | | - | - | - | |
| Bo 2003 (6) | 659 | - | In hospital mortality  OR 5.08 (95%CI 3.22-8.00) p < 0.01 | | - | - | - | | - | - | - | |
| Broslawski 1995 (48) | 45 | - | Hospital LOS  ADL-0  r = -0.05 NS  ADL-6  r = -0.27 NS | | - | - | - | | - | - | - | |
| Bruno 2022 (8) | 2359 | - | 3-month mortality  a-HR 0.84 (0.80-0.89) p < 0.01 | | - | - | - | | - | - | - | |
| Chan 2017 (11) | - | - | - | | 99  99 | V | SF-36 PCS  r = -0.06 (-)  EQ-5D utility  r = -0.10 (-) | | - | - | - | |
| Clini 2011 (15) | - | - | - | | 77  77  77 | D | FIM  r = -0.06 (-)  Kendall score quadriceps  r = 0.27 (-)  Kendall score biceps  r = 0.24 (-) | | 77 | D | **Before and after intervention**  Change in Katz Index from ICU admission to discharge  SRM 1.25 (+) | |
| Daubin 2011 (18) | 100 | - | 3-month mortality  OR 1.31 [0.91-1.86];  p = 0.36 | | - | - | - | | - | - | - | |
| Tripathy 2014 (42) | 109 | - | 28-day mortality  OR 0.20 (95%CI 0.10-0.80); p = 0.03 | | - | - | - | | - | - | - | |
| Vest 2011 (44) | - | - | - | | 110  45  110  45 | D | SF12 PCS  At 1-month post discharge  ß = -7.11  p < 0.001 (?)  12-months post discharge  ß = -10.71  p = 0.13 (?)  SF-12 MCS  At 1-month post discharge  ß = -3.02  p = 0.10 (?)  12-months post discharge  ß = 7.02  p = 0.16 (?) | | - | - | - | |
| Wu 1995 (47) | - | - | - | | 1746  1746  1746 | V | Duke Activity Status  r = 0.49 (-)  Sickness Impact Profile  r = 0.57 (+)  Prediction of poor functional outcome  AUC 0.70 (+) | | - | - | - | |
| **Pooled or summary result**  **(overall rating)** | 6 studies  n = 3337 | - | Conflicting results. Predictive of short-term mortality in the majority of studies | | 4 studies  n = 1967-2032 | 2 = V  2 = D | Majority of our results (10/12, 83%) did not support our hypotheses (-)  4 studies investigated convergent validity. Majority of results (10/12, 83%) did not support our hypotheses | | 1 study  n = 77 | 1 = D | 1 study involved the comparison of the Katz Index before and after intervention and supported our hypotheses (+) | |
| **Activities of Daily Living - IADL** | | | | | | | | | | | | |
| Bo 2003 (6) | 659 | - | In hospital mortality  OR 4.91 (95% CI 2.83-8.57)  p<0.01 | | - | - | - | | - | - | - | |
| Broslawski 1995 (48) | 43 | - | Hospital LOS  IADL-0  r = -0.05 NS  IADL-6  r = -0.27 NS | | - | - | - | | - | - | - | |
| Chan 2017 (11) | 95 | - | No hospitalisation between 6 and 12 months  OR 1.13 (0.87 – 1.47), NS | | 99  99 | V | SF-36 PCS  r = -0.46 (+)  EQ-5D utility  r = -0.38 (+) | | - | - | - | |
| Sacanella 2009 (39) | 230 | - | Univariate analysis  In hospital mortality  p = 0.10  Post-hospital mortality  p = 0.01  Cumulative mortality  p = 0.01  Patients with baseline IADL<5; HR=4.10 [95%CI 1.50-11.00]; p=0.006 | | - | - | - | | - | - | - | |
| **Pooled or summary result**  **(overall rating)** | 2 studies  n = 889 | - | Conflicting results for in-hospital mortality. Significant association with post-hospital and cumulative mortality | | 1 study  n = 99  n = 198 | 1 = V | Majority of our results (2/2, 100%) supported our hypotheses. (+)  1 study investigated convergent validity. All results (2/2, 100%) supported our hypotheses | | - | - | - | |
| **Handgrip Strength** | | | | | | | | | | | | |
| Ali 2008 (2) | 136 | - | In hospital mortality  OR 4.5 (95% CI 1.5-13.6);  p < 0.01 | | 136 | A | Muscle strength (MRC)  r = 0.64 (+)  Diagnosis of ICU-AW  sensitivity 80.6%, specificity 83.2%, NPV 92.3%, PPV 63.0%  (?) | | - | - | - | |
| Bakhru 2018 (4) | 35 | - | 1-year mortality  OR 0.95 (95% CI 0.88-1.02); p = 0.16  6-month readmissions  OR 0.96 (95% CI 0.88-1.06); p = 0.42 | | - | - | - | | - | - | - | |
| Chan 2017 (11) | 224 | - | No hospitalisation between 6-12months  OR 1.08 (95% CI 0.95 to 1.24), NS | | 224  224 | V | SF-36 PCS  r = 0.19 (-)  EQ-5D Utility  r = 0.11 (-) | |  |  |  | |
| Chan 2018 (12) |  |  |  | | 89  85 | V | DXA lean body mass at 6 months  r = 0.07 (-)  DXA lean body mass at 12 months  r = -0.15 (-) | |  |  |  | |
| Cottereau 2015 (17) | 84 | - | Prolonged weaning  AUC 0.66 | |  |  |  | | - | - | - | |
| Dos Reis 2022 (21) |  |  |  | | 122 | D | Barthel index  r = 0.57 (+) | |  |  |  | |
| Fan 2014 (22) | - | - | - | | 222  222  222 | D | Muscle strength (MRC)  r = 0.99 (+)  MIP  r = 0.99 (-)  Arm muscle area  r = 0.97 (-) | | - | - | - | |
| Lee 2012 (28) | 107 | - | ICU LOS  r = 0.06  Hospital LOS  r = 0.05  In hospital mortality  OR 1.00 (95% CI = 0.93 to 1.04); p = 0.74 | | 107 | D | Muscle strength (MRC)  r = 0.55 (+) | | - | - | - | |
| Melo 2022 (30) |  |  |  | | 142 | V | 5xSTS  r = -0.57 (+) | |  |  |  | |
| Mohamed-Husein 2017 (31) | 34 | - | ICU LOS  r = -0.19  Duration of MV  r = -0.34 | |  |  |  | | - | - | - | |
| Needham 2014 (32) |  |  |  | | 164  164  167 | V | SF-36 PF  r = 0.32 (+)  6MWT  r = 0.28 (-)  Muscle strength  r = 0.40 (-) | |  |  |  | |
| Parry, Berney 2015 (36) | 29 | - | MV duration  r = −0.30  Hospital LOS  r = −0.30 | | 29  29  29 | D | MMT 6-point diagnosis  of ICU-AW  r = 0.86 (+)  PFIT-S (awakening)  r = 0.56 (+)  PFIT-s  (ICU discharge)  r = 0.38 (+)  Diagnosis of ICU-AW  Right  AUC 0.84 (+)  Sensitivity 0.88  Specificity 0.80  PPV 0.76  NPV 0.91  Left  AUC 0.85 (+)  Sensitivity 0.96  Specificity 0.74  PPV 0.72  NPV 0.96 | |  |  |  | |
| Van Der Schaaf 2008 (43) |  |  |  | | 69 | V | Barthel index  r = 0.62 (+) | |  |  |  | |
| **Pooled or summary result** | 7 studies  n = 649 | - | Conflicting results | | 10 studies  n = 1300-1307 | 4 = V  2 = A  4 = D | 60% (12/20) of results supported our hypotheses (±)  10 studies investigated convergent validity. 56% (10/18) of results supported our hypotheses  1 study investigated divergent validity. Majority of results (2/2, 100%) supported our hypotheses | | - | - | - | |
| **GLIM** | | | | | | | | | | | | |
| Shahbazi 2021 (40) | 109 | - | Hospital LOS  OR 3.43 (95% CI 1.16-10.15); p=0.02  ICU mortality  OR 4.83 (95% CI 2.09-11.15); p<0.001 | | 109 | V | SGA  AUC 0.93 (95% CI: 0.87-0.99) (+) | | - | - | - | |
| Theilla 2021 (41) | - | - | - | | 84  84  84  84 | V | SGA  AUC 0.85 (+)  Low FFMI  r = -0.54 (+)  Phase angle  r = 0.35 (+)  PANDORA  r = 0.29 (-) | | - | - | - | |
| **Pooled or summary result**  **(overall rating)** | 1 study  n = 109 | - | Predictive of hospital length of stay and ICU mortality | | 2 studies  n = 193 | 2 = V | Majority of results (4/5, 80%) supported our hypotheses (+)  2 studies investigated convergent validity. Majority of results (3/4, 75%) supported our hypotheses | | - | - | - | |

A = adequate; a-HR = adjusted hazard ratio; AUC = area under the curve; CES-D = center for epidemiologic studies-depression scale; CI = confidence interval; D = doubtful; ES = effect size; FAC = functional ambulation category; FFMI = fat free mass index; FIM= functional independence measure; FPI = functional performance inventory; FSS-ICU = functional status score for the intensive care unit; GLIM = global leadership initiative on malnutrition; HADS = hospital anxiety and depression scale; HGS = handgrip strength; HHD = hand held dynamometry; I = inadequate; ICC = intraclass coefficient; ICU = intensive care unit; IES-R = impact of event scale-revised; LHS=left hand side; LOS = length of stay; MCS = mental component summary score; MH = mental health; MIC = minimal important change; MIP = maximum inspiratory pressure; MRCSS = Medical Research Council sum score; NA = not applicable; NS = not significant; OR = odds ratio; PANDORA = patient and nutrition derived outcome risk assessment score; PCS = physical component score; PFIT-s = physical function in ICU test; PQOL = Patrick’s perceived quality of life; RHS = right hand side; SD = standard deviation; SDC = smallest detectable change; SEM = standard error of measurement; SGA = subjective global assessment; SF-36 = short form-36 questionnaire; SPPB = short physical performance battery; STS = sit-to-stand; TUG = timed up and go; V = very good; XSFMA-F/B = extra short musculoskeletal function assessment; 6MWT = 6-minute walk test; - (dash) = not assessed

Supplementary material

*Table S7. Floor and ceiling effects*

| **Measurement Instrument** | **In Hospital** | | **ICU Recovery** | |
| --- | --- | --- | --- | --- |
|  | **Floor effect** | **Ceiling effect** | **Floor effect** | **Ceiling effect** |
| **SF-36 PF** | - | - | 3 months (14): 6%  24 months (16): 16%  1-6 years (39): 32% | 3 months (14): 20%  24 months (16): 9%  1-6 years (39): 38% |
| **SF-36 PCS** | **-** | - | 6 months (3): 3% | 6 months (3): No ceiling effect |
| **STS** | 30STS  ICU discharge (7): 15%  Hospital discharge (7): No floor effect | 30STS  ICU discharge (7): No ceiling effect  Hospital discharge (7): No ceiling effect | 5xSTS  3 months (17): 35% | **-** |
| **6MWT** | Hospital discharge (40): 40% | - | 3 months (17): 4% | **-** |
| **SPPB** | Awakening (9): 83%  ICU discharge (9): 57% | - | **-** | **-** |
| **SPPB 4m gait speed** | - | - | **-** | **-** |
| **Handgrip strength** | During ICU admission (31,34,41):  55%, 30%, 26% | - | **-** | **-** |
| **Barthel Index** | ICU discharge (10): 11% | ICU discharge (10): 1% | **-** | **-** |
| **Katz Index** | - | - | **-** | **-** |
| **IADL** | - | - | **-** | **-** |
| **GLIM** | - | - | **-** | **-** |

HGS = handgrip strength; IADL = instrumental activities of daily living; ICU = intensive care unit; SF-36 PCS = Physical component score of the short form 36; SF-36 PF = Physical functioning score of the short form 36; SPPB = Short physical performance battery; 30STS = 30 second sit-to-stand; 5xSTS = five times sit-to-stand; 6MWT = 6-minute walk test.

Supplementary material

*Table S8. Summary of Clinimetric Properties and Quality of Evidence*

| **Measurement instrument** | **Summary** | **Overall rating** | **Certainty of evidence (GRADE)** |
| --- | --- | --- | --- |
| **Structural validity** | | | |
| SF-36 PF | No studies | **-** | **-** |
| SF-36 PCS | No studies | **-** | **-** |
| STS | No studies | **-** | **-** |
| 6MWT | No studies | **-** | **-** |
| SPPB | No studies | **-** | **-** |
| Barthel Index | No studies | **-** | **-** |
| Katz Index | No studies | **-** | **-** |
| IADL | No studies | **-** | **-** |
| Handgrip strength | No studies | **-** | **-** |
| GLIM | No studies | **-** | **-** |
| **Internal consistency** | | | |
| SF-36 PF | Good to excellent internal consistency  Cronbach’s alpha range 0.93-0.96  Weighted Mean (SD) 0.94 (0.01) | Indeterminate (?)* | High  ⊕⊕⊕⊕ |
| SF-36 PCS | No studies | - | - |
| STS | No studies | - | - |
| 6MWT | No studies | - | - |
| SPPB | No studies | - | - |
| Barthel Index | Excellent internal consistency  Cronbach’s alpha 0.81 | Indeterminate (?)* | Low  ⊕⊕◯◯ |
| Katz Index | No studies | - | - |
| IADL | No studies | - | - |
| Handgrip strength | No studies | - | - |
| GLIM | No studies | - | - |
| **Reliability** | | | |
| *Test-retest* |  |  |  |
| SF-36 PF | Moderate to excellent test-retest reliability  ICC range 0.56–0.98  Weighted mean (SD) 0.86 (0.19) | Sufficient (+) | Low  ⊕⊕◯◯ |
| SF-36 PCS | No studies |  |  |
| STS | Excellent test-retest reliability  ICC 0.99 (95% CI 0.99-0.99) | Sufficient (+) | Very low  ⊕◯◯◯ |
| 6MWT | No studies | - | - |
| SPPB | No studies | - | - |
| SPPB – 4m gait speed | Excellent test-retest reliability  ICC range 0.89-0.99 | Sufficient (+) | Low  ⊕⊕◯◯ |
| Barthel Index | No studies | - | - |
| Katz Index | No studies | - | - |
| IADL | No studies | - | - |
| Handgrip strength | Excellent test-retest reliability  ICC range 0.86-0.92  Weighted Mean (SD) 0.89 (0.03) | Sufficient (+) | Very low  ⊕◯◯◯ |
| GLIM | No studies | - | - |
| *Inter-rater Reliability* |  |  |  |
| SF-36 PF | No studies | - | - |
| SF-36 PCS | No studies |  |  |
| STS | Excellent inter-rater reliability  ICC range 0.85-0.99  Pooled ICC (95% CI) 0.95 (0.77-0.99) (I^2^=98%) | Sufficient (+) | Moderate  ⊕⊕⊕◯ |
| 6MWT | No studies | - | - |
| SPPB | No studies | - | - |
| SPPB – 4m gait speed | Excellent inter-rater reliability  ICC 0.97 | Sufficient (+) | Low  ⊕⊕◯◯ |
| Barthel Index | Excellent inter-rater reliability  ICC 0.98 (95% CI 0.97-0.98) | Sufficient (+) | Low  ⊕⊕◯◯ |
| Katz Index | No studies | - | - |
| IADL | No studies | - | - |
| Handgrip strength | Excellent inter-rater reliability  ICC Range 0.89-0.98  Pooled ICC (95% CI) 0.95 (0.91-0.97) (I^2^=64%) | Sufficient (+) | Low  ⊕⊕◯◯ |
| GLIM | No studies | - | - |
| **Measurement error** | | | |
| SF-36 PF | No studies | - | - |
| SF-36 PCS | One study reported measurement error and clearly explained how calculations were performed. It provided indeterminate quality evidence due to no calculation of SDC. | Indeterminate (?) | Very low  ⊕◯◯◯ |
| STS | Three studies reported measurement error and explained how calculations were performed. They all provided indeterminate quality evidence due to no calculation of MIC. | Indeterminate (?) | Low  ⊕⊕◯◯ |
| 6MWT | One study reported measurement error and clearly explained how calculations were performed. It provided an insufficient quality of evidence due to SDC > MIC | Insufficient  (-) | High  ⊕⊕⊕⊕ |
| SPPB | One study reported measurement error and clearly explained how calculations were performed. It provided indeterminate quality evidence due to no calculation of SDC. | Indeterminate (?) | Very low  ⊕◯◯◯ |
| SPPB – 4m gait speed | One study reported measurement error and clearly explained how calculations were performed. It provided an insufficient quality of evidence due to SDC > MIC | Insufficient  (-) | Low  ⊕⊕◯◯ |
| Barthel Index | One study reported measurement error and clearly explained how calculations were performed. It provided indeterminate quality evidence due to no calculation of MIC. | Indeterminate (?) | Low  ⊕⊕◯◯ |
| Katz Index | No studies | - | - |
| IADL | No studies | - | - |
| Handgrip strength | One study reported measurement error and clearly explained how calculations were performed. It provided indeterminate quality evidence due to no calculation of MIC. | Indeterminate (?) | Very low  ⊕◯◯◯ |
| GLIM | No studies | - | - |
| **Hypotheses testing for Construct Validity** | | | |
| SF-36 PF | Majority of results (15/19, 79%) supported our hypotheses in relation to convergent and known-groups validity | Sufficient (+) | High  ⊕⊕⊕⊕ |
| SF-36 PCS | Majority of results (16/21, 76%) supported our hypotheses in relation to convergent and divergent validity | Sufficient (+) | High  ⊕⊕⊕⊕ |
| STS | Majority of results (4/4, 100%) supported our hypotheses in relation to convergent validity | Sufficient (+) | High  ⊕⊕⊕⊕ |
| 6MWT | Majority of results (22/26, 85%) supported our hypotheses in relation to convergent and divergent validity | Sufficient (+) | High  ⊕⊕⊕⊕ |
| SPPB | One result supported our hypotheses in relation to convergent validity (1/1, 100%) | Sufficient (+) | Low  ⊕⊕◯◯ |
| SPPB – 4m gait speed | Majority of results (12/15, 80%) supported our hypotheses in relation to convergent and divergent validity | Sufficient (+) | High  ⊕⊕⊕⊕ |
| Barthel Index | Majority of results (8/8, 100%) supported our hypotheses in relation to convergent validity | Sufficient (+) | High  ⊕⊕⊕⊕ |
| Katz Index | Majority of results (10/12, 83%) did not support our hypotheses in relation to convergent validity | Insufficient (-) | High  ⊕⊕⊕⊕ |
| IADL | Majority of results (2/2, 100%) supported our hypotheses in relation to convergent validity | Sufficient (+) | Moderate  ⊕⊕⊕◯ |
| Handgrip strength | 60% (12/20) of results supported our hypotheses in relation to convergent and divergent validity | Inconsistent (±) | Moderate  ⊕⊕⊕◯ |
| GLIM | Majority of results (4/5, 80%) supported our hypotheses in relation to convergent validity | Sufficient (+) | High  ⊕⊕⊕⊕ |
| **Responsiveness** | | | |
| SF-36 PF | Majority of our results (3/4, 75%) supported our hypotheses | Sufficient (+) | Moderate  ⊕⊕⊕◯ |
| SF-36 PCS | Majority of our results (5/6, 83%) supported our hypotheses | Sufficient (+) | Moderate  ⊕⊕⊕◯ |
| STS | One result (1/1, 100%) supported our hypotheses | Sufficient (+) | Low  ⊕⊕◯◯ |
| 6MWT | Majority of results (4/5, 80%) support our hypotheses | Sufficient (+) | High  ⊕⊕⊕⊕ |
| SPPB | One result (1/1, 100%) did not support our hypotheses | Insufficient (-) | Very low  ⊕◯◯◯ |
| SPPB – 4m gait speed | One result was indeterminate as unable to calculate effect sizes due to lack of data | Indeterminate (?) |  |
| Barthel Index | One result (1/1, 100%) supported our hypotheses | Sufficient (+) | Very low  ⊕◯◯◯ |
| Katz Index | One result (1/1, 100%) supported our hypotheses | Sufficient (+) | Very low  ⊕◯◯◯ |
| IADL | No studies | - | - |
| Handgrip strength | No studies | - | - |
| GLIM | No studies | - | - |

*

*

*Indeterminate rating due to no evidence available on structural validity. GLIM = global leadership initiative on malnutrition; CI = confidence intervals; IADL = instrumental activities of daily living; ICC = intra class coefficient; MIC = minimum important change; SD = standard deviation; SDC = smallest detectable change; SF-36 = Short Form-36 Questionnaire; SPPB = short physical performance battery; STS = sit-to-stand; 6MWT = 6-minute walk test

1. Abd-El-Gawad WM, Adly NN, Salem HM. Diagnostic accuracy of activities of daily living in prediction of community-acquired pneumonia outcomes in elderly patients admitted to intensive care units. J Clin Gerontol Geriatr. 2013 Dec 1;4(4):123–7.

2. Ali NA, O’Brien JMJ, Hoffmann SP, Phillips G, Garland A, Finley JCW, et al. Acquired weakness, handgrip strength, and mortality in critically ill patients. Am J Respir Crit Care Med. 2008 Aug 1;178(3):261–8.

3. Alison JA, Kenny P, King MT, McKinley S, Aitken LM, Leslie GD, et al. Repeatability of the six-minute walk test and relation to physical function in survivors of a critical illness. Phys Ther. 2012 Dec;92(12):1556–63.

4. Bakhru RN, Davidson JF, Bookstaver RE, Kenes MT, Welborn KG, Morris PE, et al. Physical function impairment in survivors of critical illness in an ICU Recovery Clinic. J Crit Care. 2018;45(buy, 8610642):163–9.

5. Baldwin C.E., Paratz J.D., Bersten A.D. Muscle strength assessment in critically ill patients with handheld dynamometry: An investigation of reliability, minimal detectable change, and time to peak force generation. J Crit Care. 2013;28(1):77–86.

6. Bo M, Massaia M, Raspo S, Bosco F, Cena P, Molaschi M, et al. Predictive factors of in-hospital mortality in older patients admitted to a medical intensive care unit. J Am Geriatr Soc. 2003 Apr;51(4):529–33.

7. Broslawski G, Elkins M, Algus M. Functional abilities of elderly survivors of intensive care. J Am Osteopath Assoc. 1995;95(12):712‐717.

8. Bruno RR, Wernly B, Flaatten H, Fjølner J, Artigas A, Baldia PH, et al. The association of the Activities of Daily Living and the outcome of old intensive care patients suffering from COVID-19. Ann Intensive Care. 2022 Mar 18;12(1):26.

9. Chan KS, Pfoh ER, Denehy L, Elliott D, Holland AE, Dinglas VD, et al. Construct validity and minimal important difference of 6-minute walk distance in survivors of acute respiratory failure. Chest. 2015 May;147(5):1316–26.

10. Chan KS, Aronson Friedman L, Dinglas VD, Hough CL, Morris PE, Mendez-Tellez PA, et al. Evaluating Physical Outcomes in Acute Respiratory Distress Syndrome Survivors: Validity, Responsiveness, and Minimal Important Difference of 4-Meter Gait Speed Test. Crit Care Med. 2016 May;44(5):859–68.

11. Chan KS, Aronson Friedman L, Dinglas VD, Hough CL, Shanholtz C, Ely EW, et al. Are physical measures related to patient-centred outcomes in ARDS survivors? Thorax. 2017 Oct;72(10):884–92.

12. Chan KS, Mourtzakis M, Aronson Friedman L, Dinglas VD, Hough CL, Ely EW, et al. Evaluating Muscle Mass in Survivors of Acute Respiratory Distress Syndrome: A 1-Year Multicenter Longitudinal Study. Crit Care Med. 2018 Aug;46(8):1238–46.

13. Chiang LL, Wang LY, Wu CP, Wu HD, Wu YT. Effects of physical training on functional status in patients with prolonged mechanical ventilation. Phys Ther. 2006;86(9):1271–81.

14. Chrispin PS, Scotton H, Rogers J, Lloyd D, Ridley SA. Short Form 36 in the intensive care unit: assessment of acceptability, reliability and validity of the questionnaire. Anaesthesia. 1997 Jan;52(1):15–23.

15. Clini EM, Crisafulli E, Antoni FD, Beneventi C, Trianni L, Costi S, et al. Functional recovery following physical training in tracheotomized and chronically ventilated patients. Respir Care. 2011;56(3):306–13.

16. Costigan FA, Rochwerg B, Molloy AJ, McCaughan M, Millen T, Reid JC, et al. I SURVIVE: inter-rater reliability of three physical functional outcome measures in intensive care unit survivors. Can J Anaesth J Can Anesth. 2019 Oct;66(10):1173–83.

17. Cottereau G, Dres M, Avenel A, Fichet J, Jacobs FM, Prat D, et al. Handgrip Strength Predicts Difficult Weaning But Not Extubation Failure in Mechanically Ventilated Subjects. Respir Care. 2015 Aug;60(8):1097–104.

18. Daubin C, Chevalier S, Séguin A, Gaillard C, Valette X, Prévost F, et al. Predictors of mortality and short-term physical and cognitive dependence in critically ill persons 75 years and older: a prospective cohort study. Health Qual Life Outcomes. 2011 May 16;9:35.

19. de Azevedo JRA, Lima HCM, Frota PHDB, Nogueira IROM, de Souza SC, Fernandes EAA, et al. High-protein intake and early exercise in adult intensive care patients: a prospective, randomized controlled trial to evaluate the impact on functional outcomes. BMC Anesthesiol. 2021 Nov 13;21(1):283.

20. Denehy L, Nordon-Craft A, Edbrooke L, Malone D, Berney S, Schenkman M, et al. Outcome measures report different aspects of patient function three months following critical care. Intensive Care Med. 2014 Dec;40(12):1862–9.

21. Dos Reis NF, Figueiredo FCXS, Biscaro RRM, Lunardelli EB, Maurici R. Psychometric Properties of the Barthel Index Used at Intensive Care Unit Discharge. Am J Crit Care Off Publ Am Assoc Crit-Care Nurses. 2022 Jan 1;31(1):65–72.

22. Fan E, Dowdy DW, Colantuoni E, Mendez-Tellez PA, Sevransky JE, Shanholtz C, et al. Physical complications in acute lung injury survivors: a two-year longitudinal prospective study. Crit Care Med. 2014 Apr;42(4):849–59.

23. Hermans G, Clerckx B, Vanhullebusch T, Segers J, Vanpee G, Robbeets C, et al. Interobserver agreement of Medical Research Council sum-score and handgrip strength in the intensive care unit. Muscle Nerve. 2012 Jan;45(1):18–25.

24. Heyland DK, Hopman W, Coo H, Tranmer J, McColl MA. Long-term health-related quality of life in survivors of sepsis. Short Form 36: a valid and reliable measure of health-related quality of life. Crit Care Med. 2000;28(11):3599–605.

25. Kaarlola A, Pettilä V, Kekki P. Performance of two measures of general health-related quality of life, the EQ-5D and the RAND-36 among critically ill patients. Intensive Care Med. 2004 Dec;30(12):2245–52.

26. Kawakami D, Fujitani S, Morimoto T, Dote H, Takita M, Takaba A, et al. Prevalence of post-intensive care syndrome among Japanese intensive care unit patients: a prospective, multicenter, observational J-PICS study. Crit Care. 2021 Feb 16;25(1):69.

27. Khoudri I, Ali Zeggwagh A, Abidi K, Madani N, Abouqal R. Measurement properties of the short form 36 and health-related quality of life after intensive care in Morocco. Acta Anaesthesiol Scand. 2007;51(2):189–97.

28. Lee JJ, Waak K, Grosse-Sundrup M, Xue F, Lee J, Chipman D, et al. Global muscle strength but not grip strength predicts mortality and length of stay in a general population in a surgical intensive care unit. Phys Ther. 2012 Dec;92(12):1546–55.

29. Melo TA de, Duarte ACM, Bezerra TS, França F, Soares NS, Brito D. The Five Times Sit-to-Stand Test: safety and reliability with older intensive care unit patients at discharge. Rev Bras Ter Intensiva. 2019;31(1):27–33.

30. de Melo TA, Silva Guimarães F, Lapa e Silva JR. The five times sit-to-stand test: safety, validity and reliability with critical care survivors’s at ICU discharge. Arch Physiother. 2022 Dec 18;13(1):2.

31. Mohamed-Hussein AAR, Makhlouf HA, Selim ZI, Gamaleldin Saleh W. Association between hand grip strength with weaning and intensive care outcomes in COPD patients: A pilot study. Clin Respir J. 2018 Oct;12(10):2475–9.

32. Needham DM, Wozniak AW, Hough CL, Morris PE, Dinglas VD, Jackson JC, et al. Risk factors for physical impairment after acute lung injury in a national, multicenter study. Am J Respir Crit Care Med. 2014 May 15;189(10):1214–24.

33. O’Grady HK, Edbrooke L, Farley C, Berney S, Denehy L, Puthucheary Z, et al. The sit-to-stand test as a patient-centered functional outcome for critical care research: a pooled analysis of five international rehabilitation studies. Crit Care Lond Engl. 2022 Jun 13;26(1):175.

34. Parry SM, Denehy L, Beach LJ, Berney S, Williamson HC, Granger CL. Functional outcomes in ICU – what should we be using? – an observational study. Crit Care Lond Engl. 2015 Mar 29;19(1):127.

35. Parry SM, Nalamalapu SR, Nunna K, Rabiee A, Friedman LA, Colantuoni E, et al. Six-Minute Walk Distance After Critical Illness: A Systematic Review and Meta-Analysis. J Intensive Care Med. 2021 Mar;36(3):343–51.

36. Parry SM, Berney S, Granger CL, Dunlop DL, Murphy L, El-Ansary D, et al. A new two-tier strength assessment approach to the diagnosis of weakness in intensive care: an observational study. Crit Care Lond Engl. 2015 Feb 26;19(1):52.

37. Puthucheary ZA, Gensichen JS, Cakiroglu AS, Cashmore R, Edbrooke L, Heintze C, et al. Implications for post critical illness trial design: sub-phenotyping trajectories of functional recovery among sepsis survivors. Crit Care. 2020 Sep 25;24(1):577.

38. Rosa RG, Dietrich C, Valle ELT do, Souza D, Tagliari L, Mattioni M, et al. The 6-Minute Walk Test predicts long-term physical improvement among intensive care unit survivors: a prospective cohort study. Rev Bras Ter Intensiva. 2021;33(3):374–83.

39. Sacanella E, Pérez-Castejón JM, Nicolás JM, Masanés F, Navarro M, Castro P, et al. Mortality in healthy elderly patients after ICU admission. Intensive Care Med. 2009 Mar;35(3):550–5.

40. Shahbazi S, Hajimohammadebrahim-Ketabforoush M, Vahdat Shariatpanahi M, Shahbazi E, Vahdat Shariatpanahi Z. The validity of the global leadership initiative on malnutrition criteria for diagnosing malnutrition in critically ill patients with COVID-19: A prospective cohort study. Clin Nutr ESPEN. 2021 Jun;43:377–82.

41. Theilla M, Rattanachaiwong S, Kagan I, Rigler M, Bendavid I, Singer P. Validation of GLIM malnutrition criteria for diagnosis of malnutrition in ICU patients: An observational study. Clin Nutr Edinb Scotl. 2021 May;40(5):3578–84.

42. Tripathy S, Mishra JC, Dash SC. Critically ill elderly patients in a developing world—mortality and functional outcome at 1 year: A prospective single-center study. J Crit Care. 2014 Jun 1;29(3):474.e7-474.e13.

43. van der Schaaf M, Dettling DS, Beelen A, Lucas C, Dongelmans DA, Nollet F. Poor functional status immediately after discharge from an intensive care unit. Disabil Rehabil. 2008;30(23):1812–8.

44. Vest MT, Murphy TE, Araujo KLB, Pisani MA. Disability in activities of daily living, depression, and quality of life among older medical ICU survivors: a prospective cohort study. Health Qual Life Outcomes. 2011 Feb 5;9:9.

45. Weinert CR, Gross CR, Kangas JR, Bury CL, Marinelli WA. Health-related quality of life after acute lung injury. Am J Respir Crit Care Med. 1997 Oct;156(4 Pt 1):1120–8.

46. Wischmeyer PE, Hasselmann M, Kummerlen C, Kozar R, Kutsogiannis DJ, Karvellas CJ, et al. A randomized trial of supplemental parenteral nutrition in underweight and overweight critically ill patients: the TOP-UP pilot trial. Crit Care Lond Engl. 2017 Jun 9;21(1):142.

47. Wu AW, Damiano AM, Lynn J, Alzola C, Teno J, Landefeld CS, et al. Predicting future functional status for seriously ill hospitalized adults. The SUPPORT prognostic model. Ann Intern Med. 1995 Mar 1;122(5):342–50.

48. Broslawski GE, Elkins M, Algus M. Functional abilities of elderly survivors of intensive care. J Am Osteopath Assoc. 1995 Dec;95(12):712–7.
